# Bone marrow–confined IL-6 signaling mediates the progression of myelodysplastic syndromes to acute myeloid leukemia

**DOI:** 10.1172/JCI152673

**Published:** 2022-09-01

**Authors:** Yang Mei, Kehan Ren, Yijie Liu, Annabel Ma, Zongjun Xia, Xu Han, Ermin Li, Hamza Tariq, Haiyan Bao, Xinshu Xie, Cheng Zou, Dingxiao Zhang, Zhaofeng Li, Lili Dong, Amit Verma, Xinyan Lu, Yasmin Abaza, Jessica K. Altman, Madina Sukhanova, Jing Yang, Peng Ji

**Affiliations:** 1 Department of Pathology, Feinberg School of Medicine, and; 2 Robert H. Lurie Comprehensive Cancer Center, Northwestern University, Chicago, Illinois, USA.; 3 School of Biomedical Sciences, Hunan University, Changsha, Hunan, China.; 4 Jiangsu Institute of Hematology, The First Affiliated Hospital of Soochow University, Soochow University, Suzhou, China.; 5 Department of Oncology, Albert Einstein College of Medicine, Montefiore Medical Center, Bronx, New York, USA.; 6 Department of Internal Medicine, Feinberg School of Medicine, Northwestern University, Chicago, Illinois, USA.

**Keywords:** Hematology, Inflammation, Cancer, Mouse models

## Abstract

Myelodysplastic syndromes (MDS) are age-related myeloid neoplasms with increased risk of progression to acute myeloid leukemia (AML). The mechanisms of transformation of MDS to AML are poorly understood, especially in relation to the aging microenvironment. We previously established an mDia1/miR-146a double knockout (DKO) mouse model phenocopying MDS. These mice develop age-related pancytopenia with oversecretion of proinflammatory cytokines. Here, we found that most of the DKO mice underwent leukemic transformation at 12–14 months of age. These mice showed myeloblast replacement of fibrotic bone marrow and widespread leukemic infiltration. Strikingly, depletion of IL-6 in these mice largely rescued the leukemic transformation and markedly extended survival. Single-cell RNA sequencing analyses revealed that DKO leukemic mice had increased monocytic blasts that were reduced with IL-6 knockout. We further revealed that the levels of surface and soluble IL-6 receptor (IL-6R) in the bone marrow were significantly increased in high-risk MDS patients. Similarly, IL-6R was also highly expressed in older DKO mice. Blocking of IL-6 signaling significantly ameliorated AML progression in the DKO model and clonogenicity of CD34-positive cells from MDS patients. Our study establishes a mouse model of progression of age-related MDS to AML and indicates the clinical significance of targeting IL-6 signaling in treating high-risk MDS.

## Introduction

Myelodysplastic syndromes (MDS) are age-related clonal myeloid neoplasms characterized by ineffective hematopoiesis. Patients with high-risk MDS have a significantly increased incidence of progression into acute myeloid leukemia (AML). The prognosis of AML developed from MDS is poor with limited treatment options. Genetically, recurrent chromosome abnormalities, including del(5q), loss of chromosome 7, or del(7q), are frequently detected in MDS patients (1, 2). The complex molecular pathophysiology of MDS is revealed by the fact that somatic mutations are found in over 40 genes, commonly including *SF3B1*, *TET2*, *ASXL1*, *DNMT3A*, and *TP53* (3). These mutations were also found in low allele frequency in apparently healthy old individuals with clonal hematopoiesis of indeterminate potential (CHIP) who had increased risk of developing MDS (4, 5). Adding to the complexity of MDS, age-related inflammatory bone marrow microenvironment is also involved in the development of the disease (6–13).

To understand the pathogenesis of MDS, many animal models have been developed to phenocopy MDS in patients. These generally include xenotransplantation models of hematopoietic cells from patients with MDS and genetically modified mouse models harboring mutations found in MDS (14). While the xenotransplantation models have their drawbacks of poor engraftment efficiency in mice, genetically modified mouse models are difficult to reflect the complexity of genetic abnormalities in MDS. Nevertheless, AML development was seen in *NUP98-HOXD13* (*NHD13*) hematopoietic-specific transgenic mice (15), mice with *NPM1* haploinsufficiency (16), mice with coexpression of *BCL-2* and mutant *NRAS* (17), *RUNX1*-mutant mice (18), and Arid4a-deficient mice (19). These models provide valuable tools for the investigation of potential therapeutic agents in treating MDS and preventing MDS to AML progression. However, the contributions of the bone marrow microenvironment in AML progression are unclear from these models.

We have previously shown that loss of *DIAPH1*, a gene located on 5q31 in humans and involved in the regulation of actin polymerization (20–22), led to an aberrant overexpression of CD14 on Gr1/Mac1–double-positive granulocytes and activation of Toll-like receptor 4 (TLR4) through pathogen-associated molecular patterns (PAMPs) and damage-associated molecular patterns (DAMPs) in mice (6). Our more recently reported study further explored the innate immune pathway and inflammation in MDS using a mouse model with concurrent deletion of mDia1 (encoded by *Diap1* in mouse) and microRNA-146a (miR-146a) (23). *MIR146A* is also located on chromosome 5q in humans and involved in the repression of the TLR/TRAF6 pathway (24–26). Studies using miR-146a–knockout (miR-146a–KO) mice show that miR-146a serves as a brake on inflammation and regulates myeloproliferation and oncogenic transformation (25). Therefore, mDia1/miR-146a double knockout (DKO) mice more closely mimic MDS patients with inflammatory bone marrow microenvironment. We demonstrated that mice with constitutive knockout of both mDia1 and miR-146a show age-related pancytopenia and are hypersensitive to aging-associated accumulation of DAMPs and PAMPs. This leads to an increased production of proinflammatory cytokines by myeloid-derived suppressor cells. Pathologic levels of these cytokines are detrimental to terminal erythropoiesis, leading to increased cell death, which provides more DAMPs and forms a positive-feedback loop to further worsen the inflammatory environment in these mice. These data together reveal a critical role of the inflammatory bone marrow environment in MDS pathogenesis.

In the current study, we found that DKO mice develop AML when moribund at the age of 12–14 months. We further reveal that bone marrow–confined IL-6 signaling plays a pivotal role in MDS to AML progression in the DKO model, which is also reflected in MDS patient data. Our study suggests that targeting the IL-6 signaling pathway would benefit high-risk MDS patients by preventing AML progression.

## Results

### mDia1 and miR-146a DKO mice progress from MDS to acute leukemia with aging.

**Figure 1:**
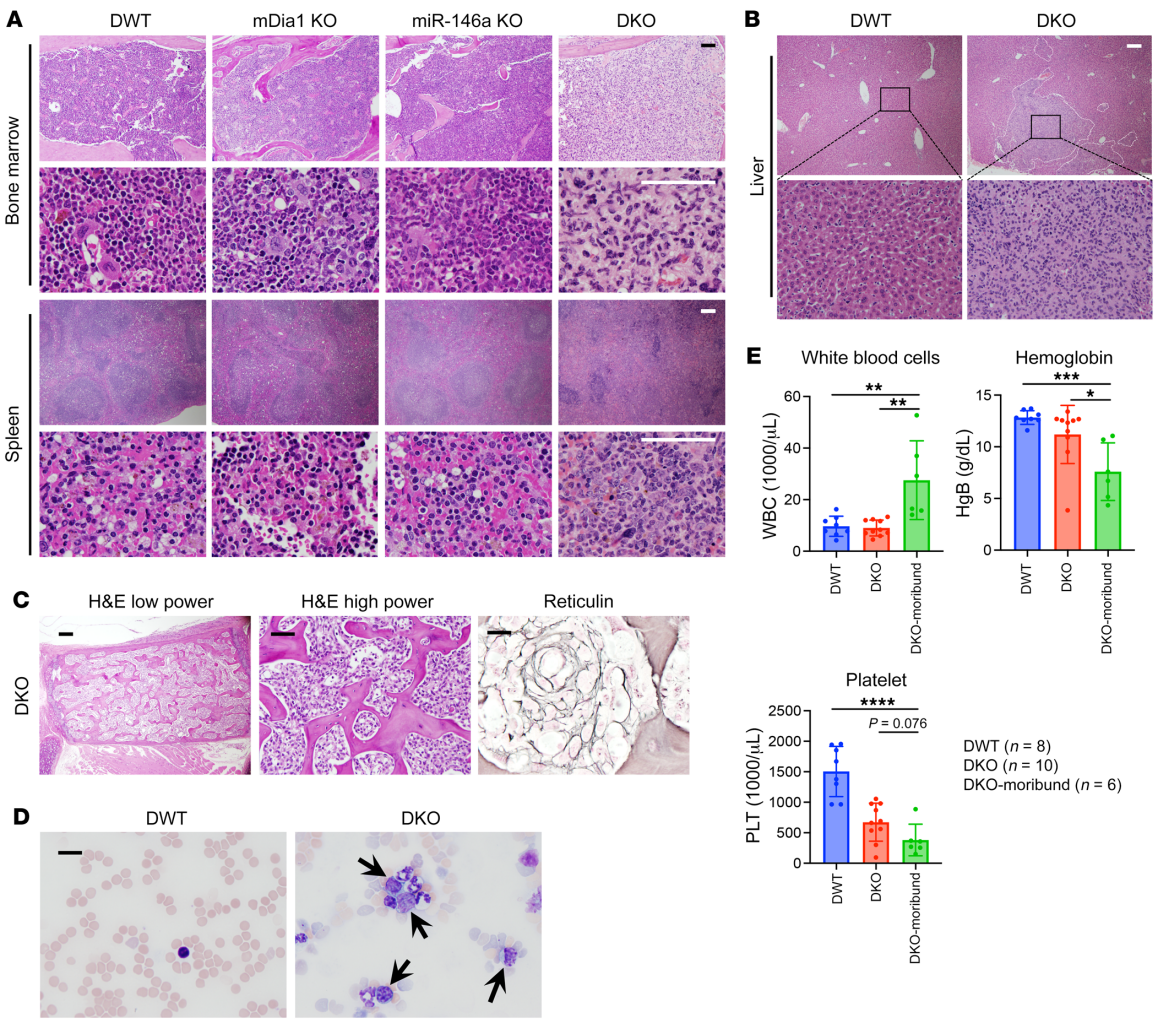
Old moribund mDia1/miR-146a DKO mice progress from MDS to acute leukemia. (**A**) Representative images of H&E staining of the bone marrow and spleens from indicated mice (12–14 months old). Scale bars: 100 μm. (**B**) Representative images of H&E staining show blasts infiltrating the livers of DKO mice (outlined) from **A**. Scale bar: 100 μm. (**C**) Representative images of the bone marrow from moribund DKO mice show osteosclerosis (H&E) and marked fibrosis (reticulin). Scale bars: 100 μm. (**D**) Wright-Giemsa staining of peripheral blood smear from moribund DKO mice and DWT control mice. Arrows indicate the blasts. Scale bar: 20 μm. (**E**) White blood cell count, hemoglobin, and platelet count in the indicated mice. The mice in these groups were 12–14 months old. Data are presented as mean ± SEM. *P < 0.05, **P < 0.01, ***P < 0.001, ****P < 0.0001; 1-way ANOVA.

*DIAPH1* and *MIR146A* are located on chromosome 5q, which is commonly deleted in patients with MDS (1, 6, 7, 27–29). Genetic abnormalities in MDS involving chromosome 5q usually show a single allele deletion. However, studies also demonstrated that many genes on the intact allele are epigenetically silenced (1, 2), which was also the case for *DIAPH1* and *MIR146A* (Supplemental Figure 1A; supplemental material available online with this article; https://doi.org/10.1172/JCI152673DS1) (27, 30). Our recently reported study demonstrated that constitutive mDia1/miR-146a DKO mice develop age-related MDS manifested as anemia, thrombocytopenia, and ineffective hematopoiesis. These mice show markedly increased inflammatory cytokines in the bone marrow and increased lethality (23). We monitored DKO mice more closely when they became moribund (12–14 months old) and dissected the mice. In contrast to the double-wild-type (DWT) and the mDia1 or miR-146a single-KO mice, the bone marrow of the majority of the moribund DKO mice (>90%) was completely replaced by monotonous blasts with marked reduction of normal bone marrow hematopoietic cells. There was also marked leukemia involvement of the spleen in that the normal splenic architecture was effaced by blasts (Figure 1A). In addition, other organ systems were infiltrated by blasts (Figure 1B). Marked osteosclerosis and fibrosis were also seen in the bone marrow of DKO mice (Figure 1C). The blasts could also be observed in circulation in these mice (Figure 1D). Consistently, the white blood cell counts in the moribund DKO mice were significantly increased (Figure 1E). Overall, the mDia1/miR-146a DKO mice progressed to acute leukemia from age-related MDS.

### Loss of IL-6 reverts the leukemic progression in DKO mice.

**Figure 2:**
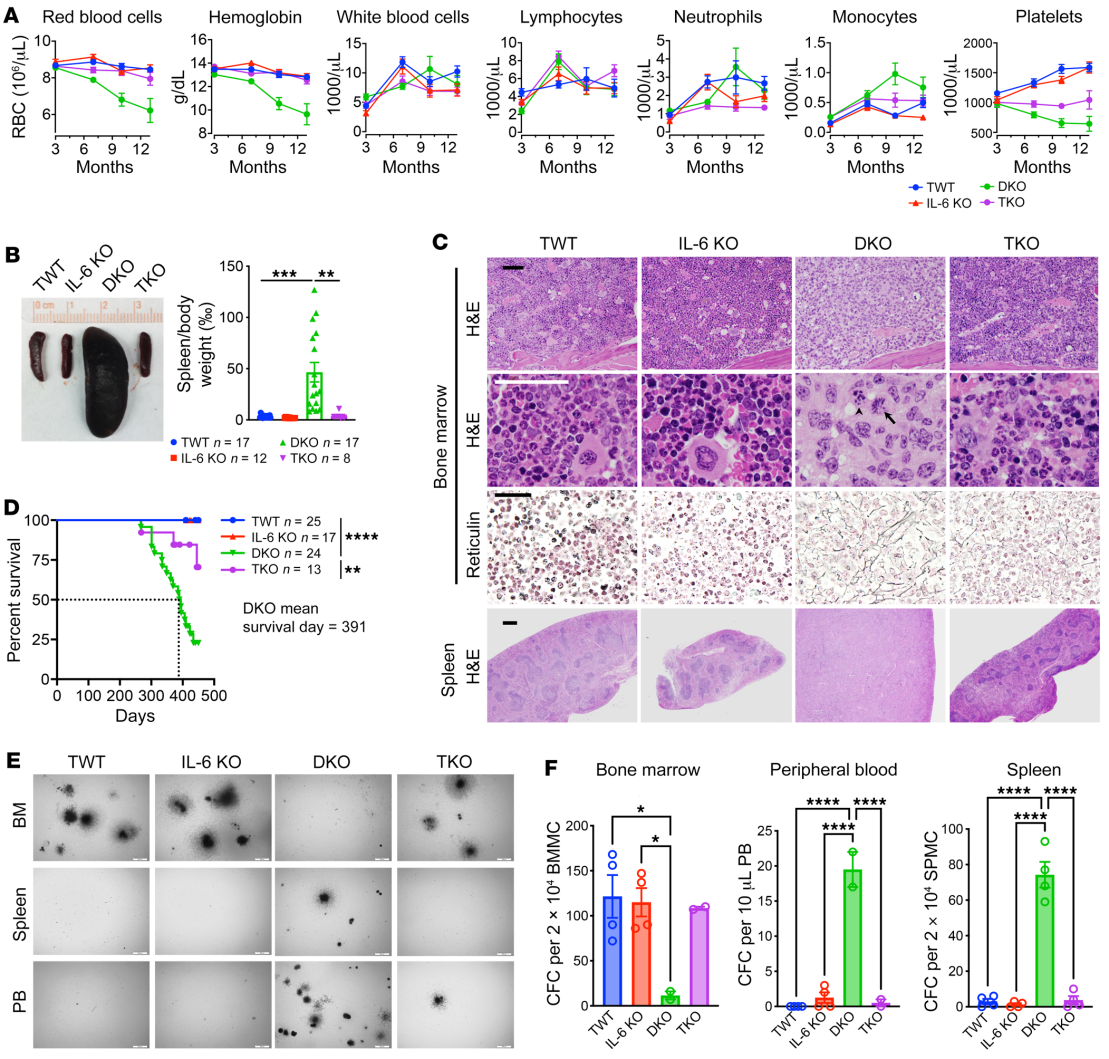
IL-6 signaling mediates MDS transformation to acute leukemia. (**A**) Complete blood cell counts of indicated mice at indicated time points. TWT, *Diap1^+/+^ Mir146a^+/+^ Il6^+/+^*, *n* = 11; IL-6 KO, *n* = 7; DKO, *Diap1^–/–^ Mir146a^–/–^ Il6^+/+^*, *n* = 16; TKO, *Diap1^–/–^ Mir146a^–/–^ Il6^–/–^*, *n* = 8. (**B**) Representative spleen images from the indicated mice (left) at 12–14 months of age. The spleen/body weight ratio was further quantified (right). (**C**) Representative histology images of bone marrow and spleen from the indicated mice in **B**. The reticulin staining reveals increased fibrosis in DKO mice. The arrow and arrowhead indicate mitotic and apoptotic cells, respectively. Scale bars: 100 μm. (**D**) Kaplan-Meier survival analysis of the indicated mice. (**E** and **F**) In vitro colony-forming unit assay of nucleated cells from the bone marrow (BM), spleen, and peripheral blood (PB) of indicated mice at 12–14 months of age. Representative colonies are shown in **E** and quantified in **F**. Scale bars: 500 μm. CFC, colony-forming cell; BMMC, bone marrow mononuclear cell; SPMC, splenic mononuclear cell. Data are presented as mean ± SEM. *P < 0.05, **P < 0.01, ***P < 0.001, ****P < 0.0001; 1-way ANOVA.

We previously demonstrated that the inflammatory bone marrow microenvironment is essential for the development of MDS in DKO mice (23). Among the inflammatory cytokines that are upregulated in DKO mice, IL-6 shows the highest fold increase in the DKO model, indicating that it could play a major role in mediating the pathogenesis of DKO mice. Therefore, we crossed the DKO mice with constitutive IL-6–KO mice and generated mDia1/miR-146a/IL-6 triple knockout (TKO) mice. These mice exhibited no detectable abnormalities at steady state when they were young, including normal complete blood count (Figure 2A). As we reported, the DKO mice started to exhibit anemia, thrombocytopenia, and monocytosis with aging. In contrast, TKO mice showed a marked reversion of these parameters (Figure 2A). When the moribund DKO mice (12–14 months old) were sacrificed and compared with the age-matched triple-wild-type (TWT), IL-6–KO, and TKO mice, we found that the marked splenomegaly in DKO mice was reverted to the normal level in TKO mice (Figure 2B). We next examined the histopathology of these mice. As expected, bone marrow trilineage hematopoiesis and spleen normal architecture were largely restored in TKO mice compared with the DKO mice, which showed complete replacement by blasts in these organs. Bone marrow fibrosis, as well as multiorgan leukemia infiltration, was also substantially reverted with IL-6 deficiency (Figure 2C and Supplemental Figure 1, B–D). More important, IL-6 deficiency significantly extended the survival of the DKO mice (Figure 2D). The replacement by blasts abolished the colony-forming capacities of the bone marrow cells in DKO mice. Instead, extramedullary hematopoiesis was evident, demonstrated by colony formation by spleen cells and circulating mononucleated cells. These changes were also reverted by the loss of IL-6 (Figure 2, E and F). Together, these results reveal that IL-6 plays a major role in mediating MDS to leukemia progression in DKO mice.

### IL-6 is critical for the progression of MDS to myelomonocytic leukemia in the DKO model.

**Figure 3:**
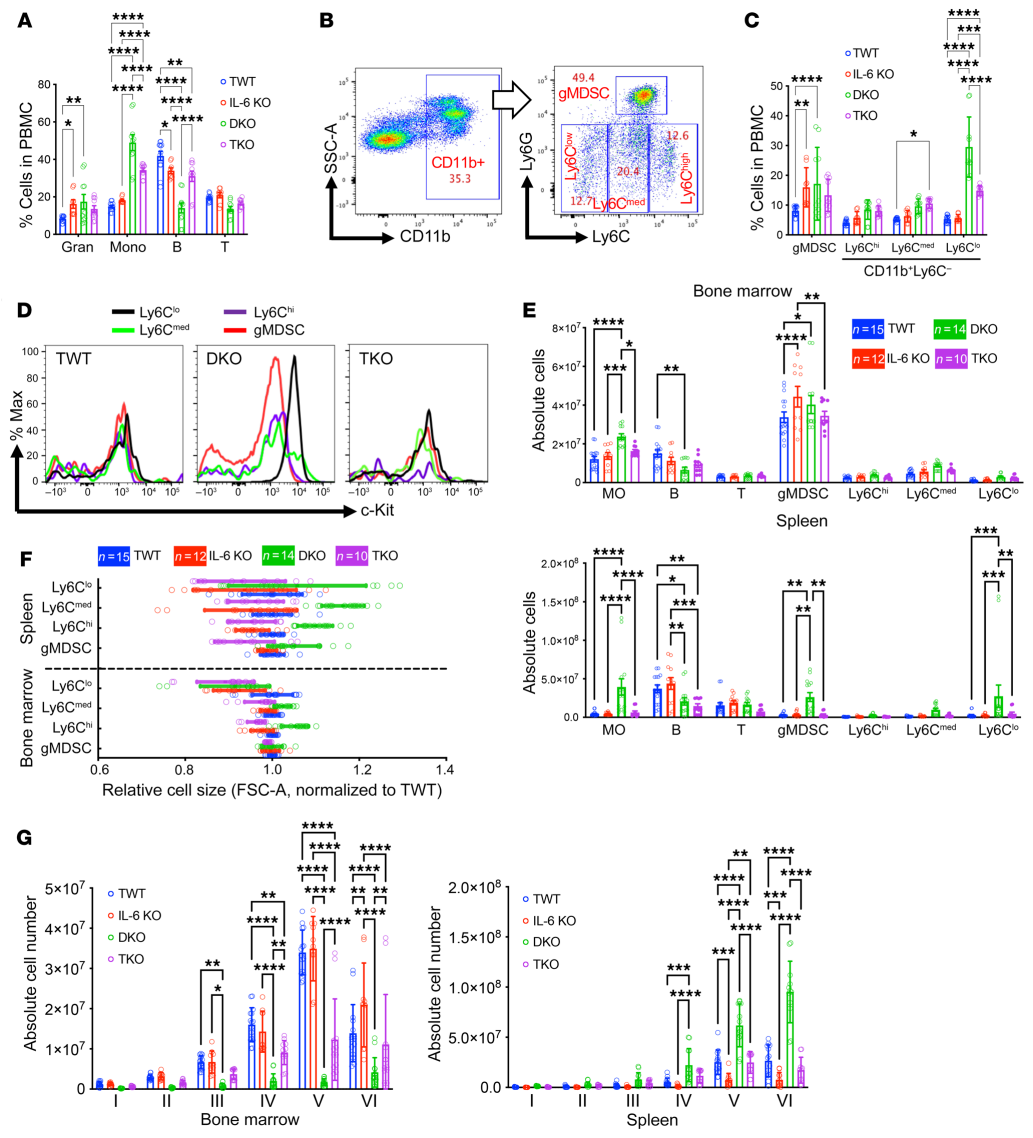
IL-6 deficiency ameliorates the defective hematopoiesis and leukemogenesis in DKO mice. (**A**) The percentages of indicated cells among the peripheral blood mononuclear cells of the indicated mice at 12–14 months of age. TWT, *n* = 12; IL-6 KO, *n* = 8; DKO, *n* = 10; TKO, *n* = 10. Gran, granulocytes, Ly6G^+^CD11b^+^; MO, monocytes, Ly6C^+^CD11b^+^; B, B cells, B220^+^; T, T cells, CD3e^+^. (**B**) Flow cytometry plots illustrating the gating strategy for MDSCs in cells from **A**. gMDSC, granulocytic MDSC. (**C**) Quantification of MDSC populations in **B**. (**D**) Flow cytometric analyses of the expression levels of c-Kit among the indicated cell populations from **C** in the indicated mice. (**E**) Absolute numbers of cells in the indicated lineages were quantified in the bone marrow and spleens from the indicated mice in **A**. (**F**) Cell size of indicated MDSCs in **E** was measured by flow cytometric forward scatter (FSC-A) and normalized to cells from the TWT group. (**G**) Absolute number of erythroid cells in various developmental stages (I–VI) from the bone marrow and spleens of the indicated mice in **C**. The stages were determined by the cell surface expression levels of CD44. Stage I, proerythroblast; stage II, basophilic erythroblast; stage III, polychromatic erythroblast; stage IV, orthochromatic erythroblast; stage V, reticulocyte; stage VI, mature erythrocyte. Data are presented as mean ± SEM. *P < 0.05, **P < 0.01, ***P < 0.001, ****P < 0.0001; 2-way ANOVA.

We next harvested the hematopoietic tissues from these mice (12–14 months old, DKO mice at moribund) to analyze the cellular compositions and types of blasts in DKO mice and how loss of IL-6 influences the transformation process. We first analyzed peripheral blood by flow cytometry before we sacrificed the mice. Consistent with the complete blood count, the DKO mice contained increased percentages of granulocytic (CD11b^+^, Ly6G^+^, and Ly6C^–^) and monocytic (CD11b^+^, Ly6G^–^, and Ly6C^+^) populations. Lymphocytes were significantly reduced (Figure 3A). We previously reported that DKO mice contained an increased number of myeloid-derived suppressor cells (MDSCs), including both granulocytic MDSCs (CD11b^+^Ly6G^+^Ly6C^lo^, same as granulocytes in this model) and monocytic MDSCs (CD11b^+^Ly6G^−^Ly6C^hi^) (23). Given this information and the presence of blasts in the peripheral blood of the DKO mice, we analyzed the CD11b^+^ myeloid populations. Among these cells, the Ly6C monocytic population was further divided into Ly6C-high, -medium, and -low cells (Figure 3B). Compared with the blood mononuclear cells from TWT and single-KO mice, those from the DKO mice contained a particularly high percentage of CD11b^+^Ly6C^lo^ cells, which are likely to be within the blast population (Figure 3C). Indeed, we found that the CD11b^+^Ly6C^lo^ cells in the DKO mice were c-Kit^+^. Similarly, loss of IL-6 reverted this phenotype (Figure 3D).

We next analyzed the bone marrow and spleens of these mice. Consistent with the findings in the peripheral blood, the overall CD11b^+^Ly6G^–^ monocytic populations, including Ly6C-high, -medium, and -low subpopulations, were substantially increased in the bone marrow of DKO mice. These monocytic cells, as well as the Ly6G^+^ granulocytic population that together constituted MDSCs, were dramatically increased in the spleen, which is consistent with the marked splenomegaly in DKO mice (Figure 3E). These cells were also relatively larger, which is consistent with their shift to immaturity when compared with WT control cells (Figure 3F). Again, loss of IL-6 nearly completely reverted these phenotypes (Figure 3, E and F). Flow cytometry assays also revealed a dramatic reduction in cells at all stages of terminal erythropoiesis in the bone marrow of DKO mice. This was associated with marked extramedullary erythropoiesis in the enlarged spleen in these mice. Loss of IL-6 largely reverted these phenotypes in erythropoiesis as well (Figure 3G and Supplemental Figure 2). Overall, these results reveal that the DKO mouse represents a model of MDS transformation to acute myelomonocytic leukemia. The blast population in the leukemia phase is predominantly monocytic c-Kit^+^ cells.

### Single-cell RNA sequencing and cytokine analyses of DKO and TKO models.

To investigate the leukemic transformation in DKO mice at the single-cell level, we sequenced 8423, 10,866, and 9013 mononuclear cells from the bone marrow of 12- to 14-month-old TWT, DKO, and TKO mice, respectively. TWT and TKO individual cells had comparable median gene numbers (TWT 1332 vs. TKO 1384) and median unique molecular identifier (UMI) transcripts (TWT 4363 vs. TKO 4591). In contrast, the DKO single cells had higher median gene number (1744) and UMI transcripts (6596). The most significant marker gene expression profiles in each cluster exhibited similarities between TWT and TKO mice, whereas DKO cells had a more unique pattern (Supplemental Figures 3–5).

**Figure 4:**
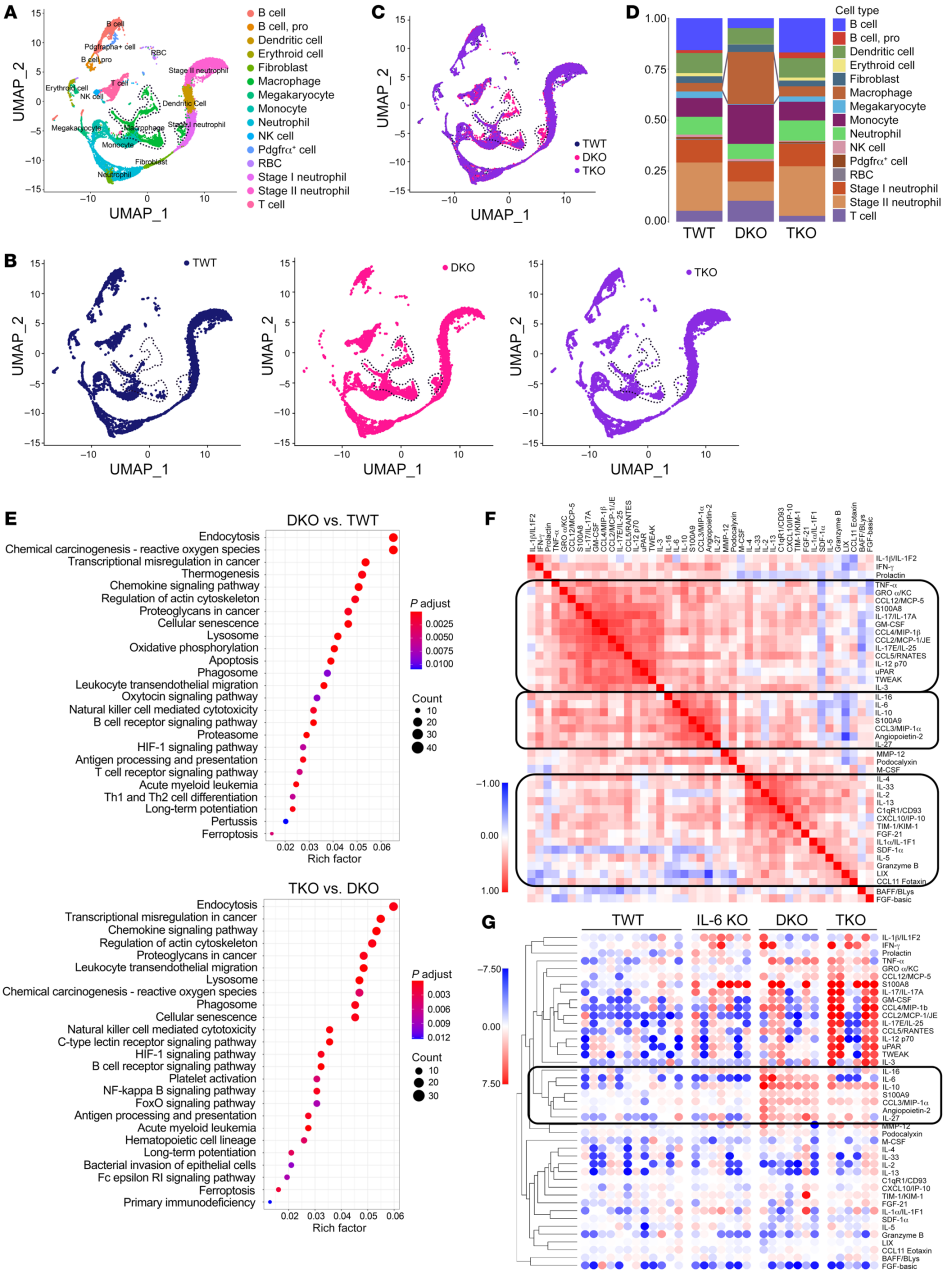
Single-cell RNA sequencing profiling reveals IL-6 signaling mediating MDS transformation to AML with monocytic differentiation. (**A**) Merged Uniform Manifold Approximation and Projection (UMAP) plots from the bone marrow of 12- to 14-month-old TWT, DKO, and TKO mice showing the distribution and overlapping of annotated cell populations. (**B**) Same as **A** except the plots are shown separately for TWT, DKO, and TKO. (**C**) Merged UMAP plots from **B** highlighting the increased cell populations. (**D**) The percentages of the annotated cell types were compared among the indicated groups of mice. (**E**) KEGG pathway enrichment analysis of differentially expressed genes from **A**–**D**. The size of each circle represents the count of genes in that pathway. The color key from blue to red represents the low to high of adjusted *P* value based on –log_10_. (**F**) Pairwise similarity analysis of selected cytokine levels across 4 groups of mice: TWT, IL-6 KO, DKO, and TKO. Darker red indicates coexpression patterns consistent within 4 groups. (**G**) Hierarchical-clustering analyses of cytokine expression profiles from the serum of indicated mice determined by multiplex ELISA. Each column represents serum from a single mouse.

Integrated analysis identified altered cell populations in the bone marrow of DKO mice, which were corrected by IL-6 deficiency in TKO mice (Figure 4, A and B). We captured a marked accumulation of cells expressing monocyte, macrophage, and T cell markers in DKO bone marrow, which were at low levels in TWT and TKO bone marrow (Figure 4, A–D). These findings are consistent with the flow cytometry data in which the immature monocytic cells represent the blast population (Figure 3). Further analyses examined the differentially expressed genes (DEGs) among all clusters in different genotypes, and Kyoto Encyclopedia of Genes and Genomes (KEGG) pathway enrichment analysis uncovered a significant enrichment of DEGs involved in various signaling pathways in cancers (Figure 4E). Using DEGs in clusters expressing macrophage markers as an example, KEGG pathway analysis of the altered genes identified many unique pathways that are upregulated in DKO cells and reverted in TKO (Supplemental Figure 6).

IL-6 was one of the highly upregulated proinflammatory cytokines in DKO mice in our previous report (23). To comprehensively reveal the changes of various inflammatory cytokines upon IL-6 depletion, we analyzed serum from the old mice (12–14 months) and performed a multiplex ELISA assay. A cytokine pairwise similarity assay identified 3 major clusters among cytokine expression patterns in all 4 groups (Figure 4F). A nonbiased hierarchically clustered heatmap analysis further confirmed clusters of cytokines overproduced in DKO mice, including IL-6. While IL-6 level was markedly reduced in TKO serum, the levels of other inflammatory cytokines remained unexpectedly high (Figure 4G). These data indicate that IL-6 is pivotal in driving the progression from MDS to AML in this mouse model.

### The DKO leukemia model is transplantable.

**Figure 5:**
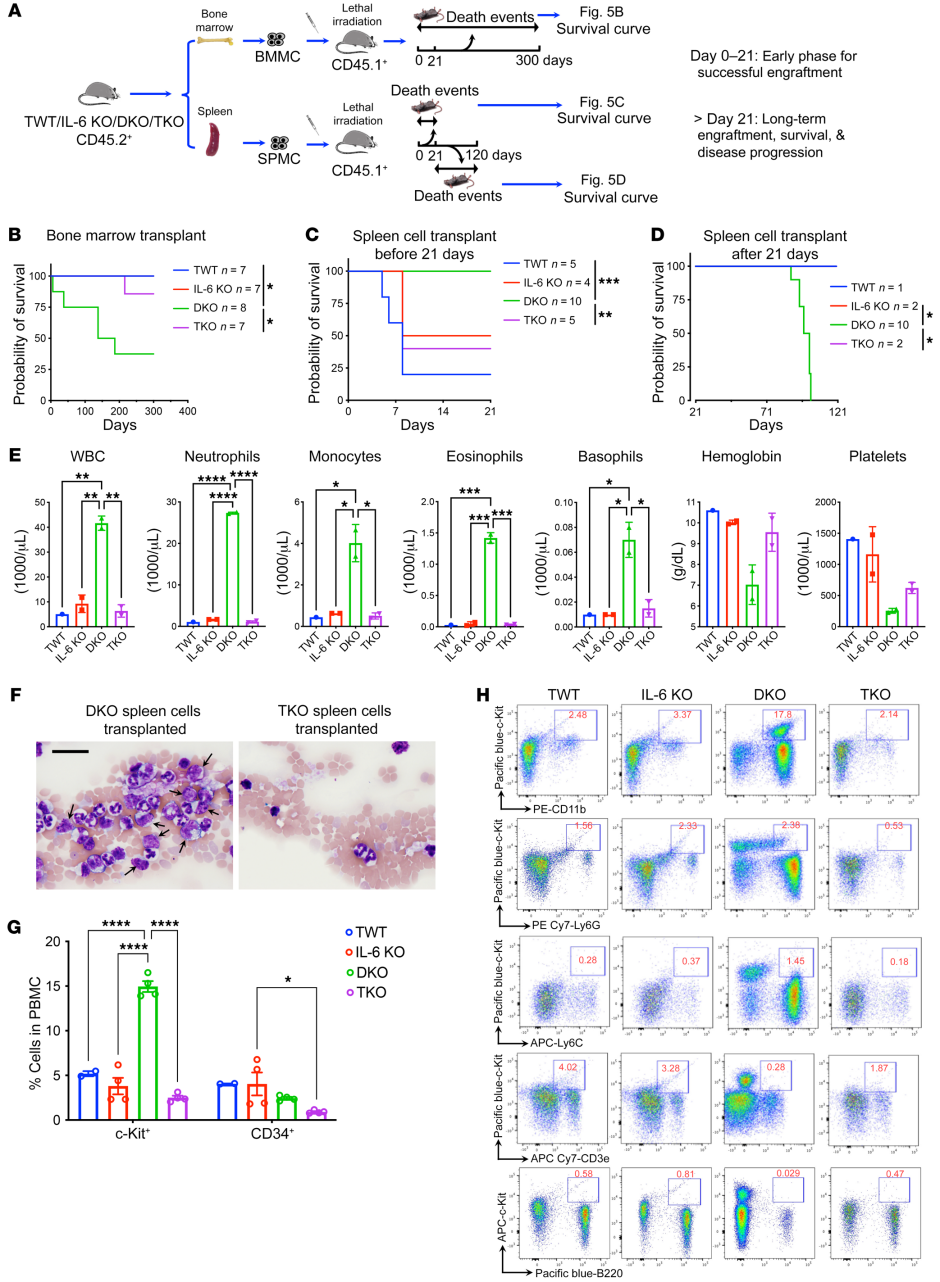
IL-6 deficiency abolishes the transplantation abilities of leukemia-initiating cells. (**A**) Schematic diagram of the transplantation strategies in **B**–**D**. (**B**) Kaplan-Meier survival analyses of CD45.1^+^ mice subjected to transplantation of 2 × 10^6^ bone marrow mononuclear cells from the indicated mice. Both the recipient and donor mice were approximately 2 months old at transplantation. (**C** and **D**) Same as **B** except 2 × 10^7^ splenic mononuclear cells from moribund DKO mice or age-matched wild-type counterparts were used as donor cells. Survival data before (**C**) and after (**D**) 21 days of transplantation are shown. (**E**) Complete blood counts of the recipient mice in **D** when the mice were 12 weeks post-transplantation. (**F**) Wright-Giemsa staining of peripheral blood smear of mice in **E**. Arrows indicate blasts. Scale bar: 20 μm. (**G**) Flow cytometric analysis evaluated stem cell surface marker expression in peripheral blood from mice in **E**. (**H**) Representative flow cytometric profiling of c-Kit^+^ cells in peripheral blood from mice in **E**. Data are presented as mean ± SEM. *P < 0.05, **P < 0.01, ***P < 0.001, ****P < 0.0001; 1-way ANOVA.

We previously reported that WT recipient mice with transplanted DKO bone marrow cells developed MDS with age-related hematologic phenotypes similar to those in DKO mice. These mice succumbed to the disease with aging (23). We performed similar transplantation experiments with the addition of TKO and IL-6–KO groups (Figure 5A). Indeed, loss of IL-6 significantly reverted the age-related lethality in this model (Figure 5B). In these assays, the donor cells were from younger mice, since bone marrow cells in leukemia-phase DKO mice were difficult to obtain owing to fibrosis. To model direct leukemia cell engraftment in the recipient mice, we purified spleen cells from moribund DKO mice and their age-matched counterparts in other groups. The spleen cells were then transplanted into lethally irradiated young WT mice (Figure 5A). Consistent with the data showing that the majority of colony-forming hematopoietic stem and progenitor cells were in the bone marrow (Figure 2E), spleen cells from TWT, IL-6–KO, and TKO mice were ineffective in engrafting the recipient mice. However, those that survived stayed alive. In contrast, all mice with transplanted splenic cells from leukemic DKO donor mice survived the initial post-transplantation stage but became rapidly lethal shortly after (Figure 5, C and D).

The moribund recipient mice with transplanted leukemic DKO splenic cells showed marked leukocytosis (including all myeloid lineages), anemia, and thrombocytopenia (Figure 5E). Many circulating blasts were readily identified and expressed c-Kit (Figure 5, F and G). We further analyzed the c-Kit^+^ cells using flow cytometry and found that most of these cells were CD11b^+^ but negative for both Ly6G and Ly6C, demonstrating their immaturity (Figure 5H).

To further demonstrate that the transplantable leukemia was derived from the blast population, we purified c-Kit^+^ cells from the spleens of 12-month-old DKO mice and transplanted them into 5-month-old lethally irradiated recipient mice. For the control group, we used bone marrow c-Kit^+^ cells from age-matched wild-type littermates, since there are few c-Kit^+^ cells in the spleens of these mice. As expected, the recipient mice with transplanted DKO splenic c-Kit^+^ cells developed leukemia within 5 months after transplantation with phenotypes similar to those of the moribund DKO mice (Supplemental Figure 7, A and B).

### IL-6 receptor and soluble IL-6 receptor are increased in the bone marrow of patients with high-risk MDS.

**Figure 6:**
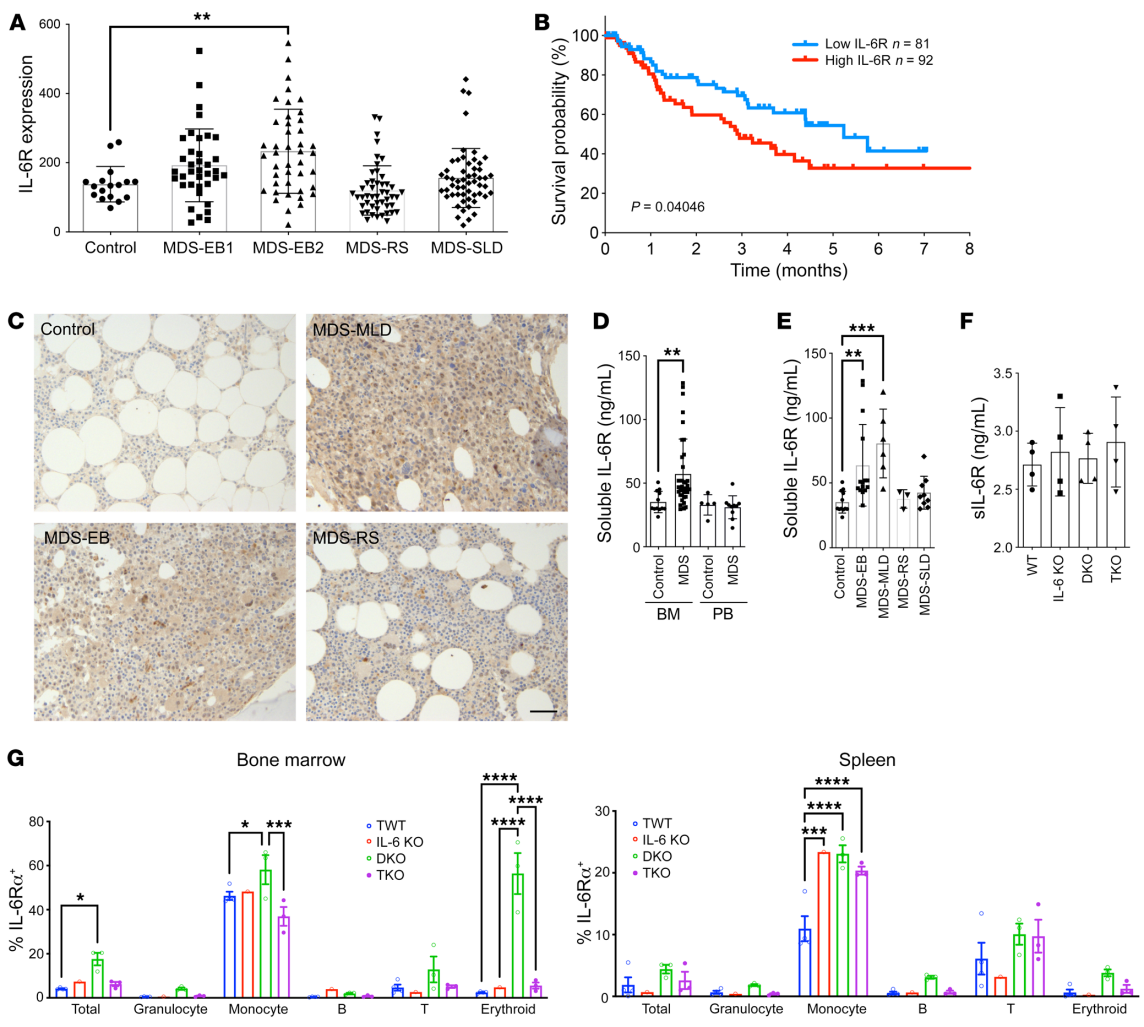
IL-6R and sIL-6R are increased in the bone marrow of patients with high-risk MDS. (**A**) IL-6 receptor (IL-6R) mRNA levels were examined from a gene expression profiling data set in CD34^+^ hematopoietic progenitor cells from patients with indicated MDS subtypes. Control, *n* = 17; MDS-EB1, MDS with excess blast-1, *n* = 37; MDS-EB2, MDS with excess blast-2, *n* = 43; MDS-RS, MDS with ring sideroblasts, *n* = 48; MDS-SLD, MDS with single-lineage dysplasia, *n* = 55. (**B**) Kaplan-Meier analysis of overall survival in MDS patients with high or low expression levels of IL-6R from **A**. (**C**) Representative images of immunohistochemical staining of IL-6R in bone marrow biopsies from patients with indicated MDS subtypes. Scale bar: 100 μm. (**D**) ELISA analyses of soluble IL-6R (sIL-6R) levels in bone marrow (BM) aspirate or peripheral blood (PB) serum from control and MDS patients in a separate cohort from **A**. BM control, *n* = 12; BM MDS, *n* = 33; PB control, *n* = 5; PB MDS, *n* = 10. (**E**) ELISA analysis of sIL-6R of bone marrow aspirate from different subtypes of MDS patients in **D**. Control, *n* = 12; MDS-EB, *n* = 15; MDS-MLD, MDS with multilineage dysplasia, *n* = 6; MDS-RS, *n* = 3; MDS-SLD, *n* = 9. (**F**) ELISA analysis of sIL-6R in serum of the indicated mice at 12 months old. *n* = 4 in each group. (**G**) Flow cytometric analyses of IL-6R expression on the cell surface of various cell lineages from the indicated mice at 12 months old. Data are presented as mean ± SEM. *P < 0.05, **P < 0.01, ***P < 0.001, ****P < 0.0001; 1-way ANOVA (**A**, **E**, and **F**), 2-way ANOVA (**G**), and 2-tailed Student’s *t* test (**D**).

The pivotal roles of IL-6 in mediating MDS to AML progression in the DKO mouse model prompted us to investigate IL-6 signaling in human patients with MDS. IL-6 is well known to be upregulated in MDS (31–34). Through its classic pathway, IL-6 binds to cell surface IL-6 receptor (IL-6R) and gp130 to trigger downstream signaling. This pathway is believed to be involved in the protective and regenerative functions of IL-6. On the other hand, the proinflammatory functions of IL-6 are mostly mediated through its trans-signaling pathway in which IL-6 and soluble IL-6R (sIL-6R) complex bind to the ubiquitously expressed gp130 in many different cell types (35–37). The expression levels of IL-6R in MDS are unclear. We first analyzed IL-6R levels in CD34^+^ hematopoietic progenitor cells in a published data set (38). We found that cells from low-risk MDS patients, including MDS with single-lineage dysplasia and MDS with ring sideroblasts, did not show differences in IL-6R mRNA expression compared with those from healthy control patients. In contrast, IL-6R was significantly upregulated in cells from patients with high-risk MDS, especially in MDS with excess blast-2 (MDS-EB2) (Figure 6A). Patients with high IL-6R expression also had a lower survival rate than those with low expression (Figure 6B). Consistent with these data, immunohistochemical stains in the bone marrow revealed a significant upregulation of IL-6R in most of the bone marrow cells in high-risk MDS compared with control-group individuals and patients with low-risk MDS (Figure 6C).

Unlike the relatively restricted expression of IL-6R, gp130 is expressed on most cell types that could mediate IL-6 signaling when IL-6 binds to a soluble form of IL-6R. Therefore, we analyzed sIL-6R in patients with various subtypes of MDS. Indeed, we found a heterogeneous but significantly increased level of bone marrow sIL-6R in MDS compared with the control group. Interestingly, the increase in sIL-6R was not observed in the serum in MDS patients (Figure 6D). Similar to the cell surface IL-6R expression patterns, the levels of sIL-6R were significantly upregulated in the bone marrow of patients with high-risk MDS, but not in low-risk subtypes (Figure 6E). We next determined whether we could observe the same phenotypes in our mouse models. As in patient serum, there were no statistically significant differences in sIL-6R levels among various groups of mice in blood (Figure 6F). We could not obtain an adequate amount of bone marrow aspirate in the DKO mice, owing to marked fibrosis. Therefore, we performed flow cytometric assays on different lineages of hematopoietic cells in the bone marrow and spleen of these mice. As expected, surface IL-6R levels were significantly increased in DKO mice (Figure 6G). The level of surface IL-6R was especially high in the bone marrow erythroid cells in DKO mice, which was unexpected since the majority of the IL-6R–expressing cells were monocytic cells in WT mice. These upregulations of surface IL-6R were normalized in TKO mice. Together, these data indicate an important role of IL-6 signaling in MDS progression to AML in both human MDS and DKO mouse models.

### Targeting IL-6 signaling ameliorates MDS to AML progression in DKO model.

**Figure 7:**
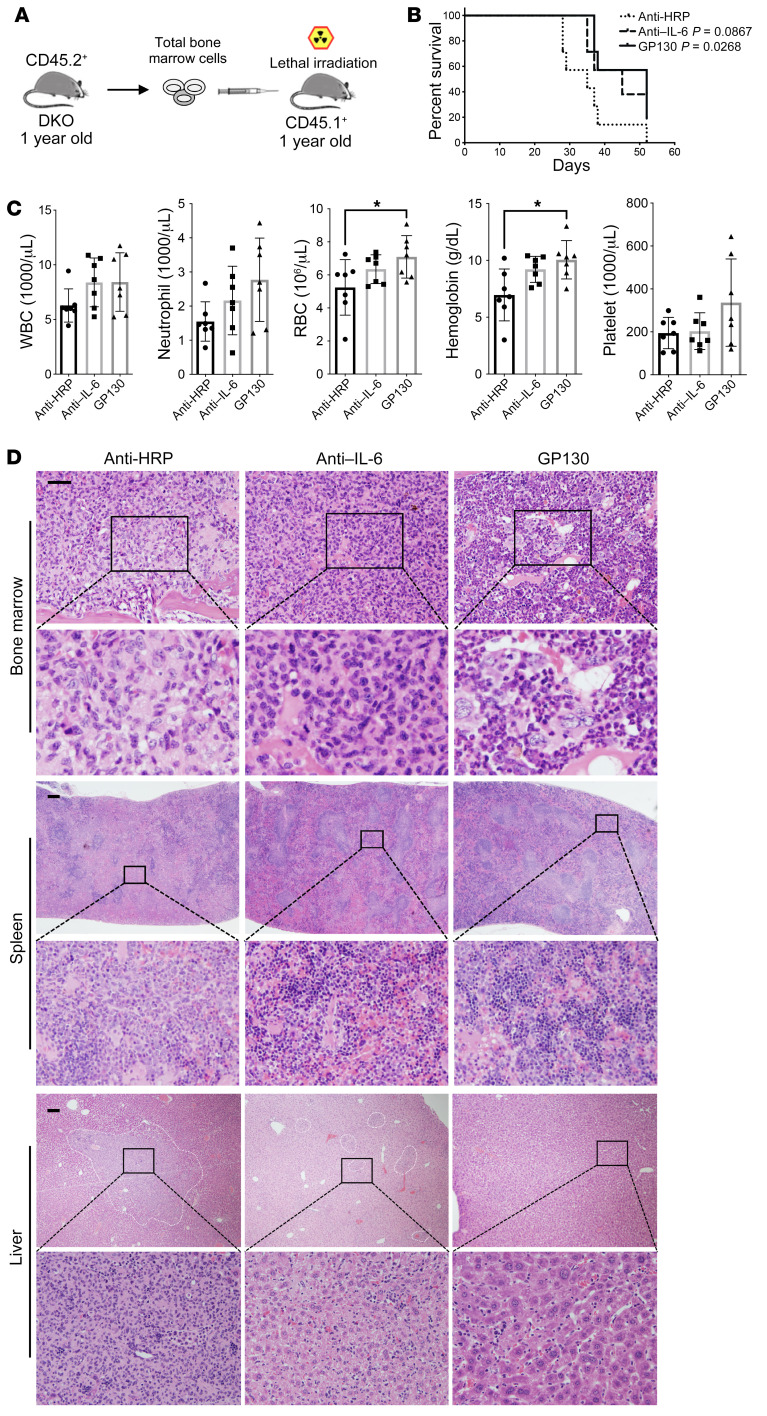
Targeting IL-6 signaling ameliorates MDS to AML progression in DKO model. (**A**) Schematic illustration of bone marrow transplantation. 5 × 10^6^ bone marrow cells from 1-year-old DKO mice were transplanted into lethally irradiated 1-year-old CD45.1^+^ recipient mice. (**B**) Kaplan-Meier survival analysis of the old CD45.1^+^ recipient mice subjected to transplantation of bone marrow cells from 1-year-old DKO mice as illustrated in **A** and treated with the indicated reagents once per week. *n* = 7 in each group. (**C**) Complete blood cell counts of the mice from **B** given the indicated reagents 1 month after treatment. Data are presented as mean ± SEM. *P < 0.05; 1-way ANOVA. (**D**) Representative H&E staining of the bone marrow, spleen, and liver from the mice in **B**. Scale bars: 100 μm.

Given the critical roles of IL-6 signaling in the progression of MDS to AML, we reasoned that inhibition of this pathway would ameliorate the phenotypes of the DKO mice. To test this, we purified total bone marrow cells from 12-month-old DKO mice that were still in the MDS stage and transplanted them into 12-month-old lethally irradiated recipient mice (Figure 7A). Through this strategy, we would be able to model the MDS to AML progression in a relatively short period with efficient initial engraftment compared with the use of AML-stage splenic cells as donors (Figure 5). We treated these mice with anti–mouse IL-6 antibodies or recombinant mouse gp130 Fc chimera proteins and compared them with control-group mice treated with anti–horseradish peroxidase (anti-HRP) mouse IgG isotype. We first confirmed the efficacies of the IL-6 antibody and gp130 Fc chimera protein in downregulating IL-6 signaling in mouse bone marrow cells (Supplemental Figure 8A). The mice in the control group developed lethal AML rapidly after transplantation within 2 months. Treatment of the mice with anti–IL-6 antibody, and specifically gp130 Fc, significantly extended survival (Figure 7B). We sacrificed these mice when the control-group mice became moribund (40 days after transplant). The mice treated with anti-HRP IgG isotype showed pancytopenia and replacement of normal hematopoiesis by blasts in the bone marrow. The normal architecture of the spleen was also effaced by blasts. The liver showed marked blast infiltration. These phenotypes were ameliorated with IL-6 antibody treatment, and particularly with gp130 Fc, in this model (Figure 7, C and D).

To determine whether inhibition of IL-6 signaling at a younger age could prevent the development of MDS in the DKO mice, we treated 5-month-old WT recipient mice with transplanted bone marrow cells from 5-month-old DKO mice chronically with anti–IL-6 antibody or gp130 Fc chimera protein. We found partial reversion of the MDS phenotypes at 1 month after treatment but loss of efficacy when the mice were tested 4 months after treatment (Supplemental Figure 8, B and C). These results further support the critical role of increased IL-6 signaling during MDS to AML progression, but not at the early stage of MDS development.

### Anti–IL-6R antibody reduces cell proliferation and clonogenicity in MDS patient cells.

**Figure 8:**
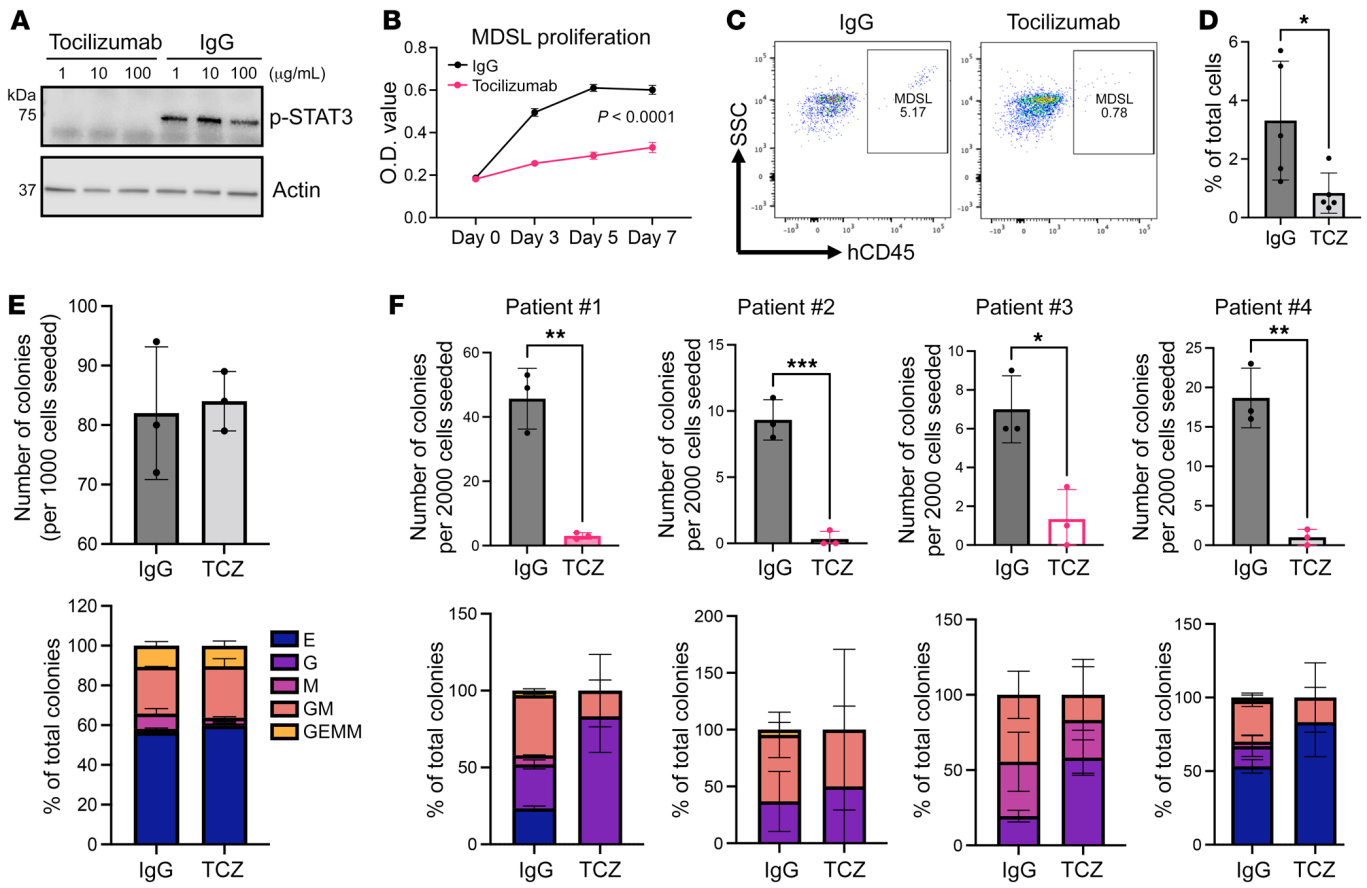
Tocilizumab reduces cell proliferation and colony formation in MDS patient cells. (**A**) Cultured MDSL cells were treated with tocilizumab or IgG for 1 hour at indicated concentrations. Cells were then challenged with human recombinant IL-6 (10 ng/mL) for 15 minutes followed by Western blot assay of p-STAT3. Actin was used as a loading control. See complete unedited blots in the supplemental material. (**B**) 1 × 10^4^ MDSL cells per well were seeded in a 96-well plate on day 0 in MDSL culture medium with 50 μg/mL tocilizumab or 50 μg/mL human IgG control. Relative cell number was assessed with CCK-8 reagent at indicated time points. (**C**) 1 × 10^6^ MDSL cells were transplanted into sublethally irradiated NSG recipient mice. Ten days after transplantation, mice were subjected to weekly tocilizumab (TCZ) or human IgG (8 mg/kg) by i.p. administration. Engraftment was evaluated 60 days after transplantation via flow cytometry assays of hCD45^+^ mononuclear cells in the peripheral blood. *n* = 5 in each group. (**D**) Quantification of the percentage of hCD45^+^ cells in **C**. (**E** and **F**) Colony-forming unit (CFU) assays in normal (**E**) and high-risk MDS patient (**F**) bone marrow–derived CD34^+^ cells. 1 × 10^3^ normal (**E**) or 2 × 10^3^ patient CD34^+^ cells (**F**) were seeded in MethoCult medium supplemented with human IgG or tocilizumab (50 μg/mL) on day 0. The number of colonies was assessed on day 14. Triplicate assay colonies were independently identified by 2 individuals. Data are presented as mean ± SD. *P < 0.05, **P < 0.01, ***P < 0.001; 2-tailed Student’s *t* test. E, G, M, GM, and GEMM represent BFU/CFU-E, CFU-G, CFU-M, CFU-GM, and CFU-GEMM, respectively, in both **E** and **F**.

To further investigate the role of IL-6 signaling in the progression of MDS to AML in patients, we first used MDSL cells. These cells were originally derived from an MDS patient and maintain the potential to engraft immunocompromised NOD/SCID-IL2Rγ (NSG) mice (39). We treated MDSL cells with tocilizumab, a monoclonal antibody against IL-6R that is clinically used to treat rheumatoid arthritis. This led to a significant reduction of p-STAT3 in vitro (Figure 8A), decreased cell proliferation (Figure 8B), and partial induction of cell death (Supplemental Figure 9A). We then treated the NSG mice with transplanted MDSL cells with tocilizumab and found significantly reduced MDSL engraftment (Figure 8, C and D). Tocilizumab also reduced the spleen weight that was increased as a result of MDSL infiltration in these mice (Supplemental Figure 9B).

To evaluate the effects of tocilizumab in primary cells from MDS patients, we purified a bone marrow CD34^+^ blast population that contained hematopoietic stem and progenitor cells from 4 patients with high-risk MDS. These patients harbored cytogenetic abnormalities and somatic mutations that are commonly seen in myeloid neoplasms (Supplemental Table 1). Tocilizumab did not affect colony expansion or composition in normal bone marrow CD34^+^ cells in an in vitro colony assay (Figure 8E). The colonies derived from MDS CD34^+^ cells expanded less robustly compared with their normal counterparts and were myeloid skewed with fewer erythroid colonies. In contrast to the normal cells, the colony numbers were markedly reduced when tocilizumab was applied in MDS-derived CD34^+^ cells (Figure 8F). Together, these results reveal that anti–IL-6R antibody is effective in reducing cell proliferation and colony formation in MDS patient cells.

## Discussion

Approximately 20%–30% of patients with MDS progress to AML (40). The prognosis after AML progression is dismal with limited treatment options. This unmet medical need necessitates the development of novel therapies to block or delay progression (14, 41). In this study, the mDia1/miR-146a DKO mice represent one of the first models that phenocopy MDS to AML progression induced by the aging bone marrow inflammatory microenvironment. Through this model, we demonstrate that IL-6 and its signaling pathway play a pivotal role in mediating disease progression.

In our previous study, we found severe anemia in DKO mice that could cause mortality. However, the leukemic transformation in DKO mice occurred within a relative short period prior to death, which led to a missed diagnosis of leukemia in the moribund mice in the previous study. In the current work, we examined the bone marrow and blood more closely, especially in moribund mice. Leukemia transformation was discovered in most of the moribund mice we investigated. In this respect, the phenotypes of the moribund DKO mice closely mimic those of patients whose high-risk MDS is transformed to AML. The prognosis in these transformed patients is also dismal, with death often occurring within a year of secondary AML diagnosis (2, 42). This period reflects a rapid lethality in the DKO model when the animals are transformed.

The dramatic rescue effects of IL-6 deficiency on AML progression in DKO mice are unexpected since multiple inflammatory cytokines are upregulated. Moreover, loss of IL-6 in these mice does not significantly reduce the levels of other cytokines, which indicates that IL-6 could function downstream or independently of most of these cytokines. Indeed, studies have shown that IL-1β induces IL-6 through a PI3K-dependent pathway (43). IL-1β is a critical mediator of the inflammatory responses and is downstream of the NLRP3 inflammasome, which is reported to function as a driver of MDS phenotype (44). The level of IL-1β is unchanged in TKO mice compared with their DKO counterparts. The roles of IL-1β and NLRP3 in the pathogenesis of the DKO model remain to be determined. Furthermore, it is likely that other cytokines, especially TNF-α, which is also highly upregulated in DKO mice (23), could also play critical roles in parallel with IL-6.

IL-6 is known to be oversecreted in many hematologic malignancies (45), including MDS (31, 32). While the pathophysiology of IL-6 signaling in several lymphoid neoplasms has been well studied (46–49), the roles of IL-6 in MDS remain observational. A phase II, randomized, double-blind multicenter study comparing anti–IL-6 effects with placebo in anemic patients with International Prognostic Scoring System low- or intermediate-1–risk MDS showed no reduction in red blood cell transfusions in transfusion-dependent patients (50). However, the study was done without knowledge of IL-6 levels in the patients. Also, high-risk MDS patients were not included. It is also unclear whether the serum or bone marrow levels of IL-6 and IL-6R are increased in different subtypes of MDS.

Consistent with this clinical study, we found that IL-6 plays less important roles in the early stage of MDS in our DKO model. This conclusion is based on the following observations. First, the MDS phenotypes remain in TKO mice. For example, TKO mice continued to have thrombocytopenia, reduced survival, and increased immature monocytic cells compared with their age-matched WT counterparts. Second, chronic treatment of young DKO mice with anti–IL-6 agents failed to revert MDS phenotypes, albeit these mice may develop resistance to IL-6 antibody. Nevertheless, a definitive answer to the question of the role of IL-6 in MDS initiation would require a genetic model in which IL-6 is deleted hematopoietic-specifically and temporally controlled in the DKO mice. It is notable that the efficacy of anti–IL-6 treatment in the DKO mice was less dramatic than that of the genetic depletion of IL-6, possibly because of the less effective downregulation of IL-6 signaling by these agents. In this respect, genetic approaches will be more important in future studies. It should also be noted that gp130 may also influence other cytokines to show a better efficacy than anti–IL-6 antibody in this model.

In the current study, while an increased level in the serum of patients with MDS was not observed, sIL-6R was highly upregulated in the bone marrow aspirate solution of MDS patients, which highlights the significance of the inflammatory bone marrow microenvironment in the pathogenesis of MDS (12, 51). Notably, sIL-6R levels in the bone marrow did not show differences between the control group and low-risk MDS. The difference became significant in the high-risk MDS groups, which further underlines the roles of IL-6 signaling in the progression of MDS to AML. Consistently, treatment of old DKO mice with anti–IL-6 agents significantly ameliorated AML progression and extended their survival. More important, tocilizumab markedly reduced the engraftment of a human MDS cell line in a xenograft model. Tocilizumab also significantly reduced clonogenicity in primary CD34^+^ cells from high-risk MDS patients. These results indicate that therapeutic management of patients with high-risk MDS, especially those with high bone marrow levels of sIL-6R, through intervention via the IL-6 signaling pathway, such as using tocilizumab, could be beneficial by reducing the progression to AML.

## Methods

### Animals.

The mDia1/miR-146a DKO mice have been described previously (23). In brief, *Mir146a^–/–^* mice in a C57BL/6 background purchased from The Jackson Laboratory (stock 016239) were crossed with mDia1-deficient mice to generate *Diap1^–/–^ Mir146a^–/–^* mice (DKO mice) (6). To generate *Diap1^–/–^ Mir146a^–/–^ Il6^–/–^* TKO mice, IL-6–KO mice purchased from The Jackson Laboratory (stock 002650) were crossed with DKO mice. CD45.1 congenic mice were purchased from Charles River Laboratories (B6-LY-5.12/Cr, strain code 564).

### Reagents.

Detailed reagent information is listed in Supplemental Table 2.

### Patient database and survival data and correlation with IL-6R gene expression.

Gene expression data from 183 MDS CD34^+^ samples and 17 controls were obtained from the NCBI’s Gene Expression Omnibus (GEO) database (GSE19429) (38) and correlated with disease subtypes and survival.

### Bone marrow transplantation.

Bone marrow transplantation was performed as described previously (6, 52, 53). Briefly, mouse total bone marrow cells were collected, followed by red blood cell lysis (Invitrogen 00-4333-57). Lethally irradiated (10 Gy) recipient mice were injected retro-orbitally with approximately 2 × 10^6^ donor bone marrow cells. The recipient mice were then fed with water containing antibiotics for 2 weeks. Complete blood cell counts (Hemavet 950, Drew Scientific) and flow cytometric analysis (BD FACSCanto II) of the peripheral blood were performed at different time points after transplantation to assess chimeras and engraftment.

For transplantation of c-Kit^+^ cells, splenic c-Kit^+^ cells from 12-month-old DKO mice and bone marrow c-Kit^+^ cells from 12-month-old WT C57BL/6 mice were isolated using c-Kit (CD117) MicroBeads (Miltenyi Biotec) according to the manufacturer’s instructions. Five-month-old CD45.1^+^ C57BL/6 recipient mice (Charles River Laboratories) were lethally irradiated (10 Gy), and 1 × 10^5^ c-Kit^+^ cells were then transplanted through retro-orbital injection.

### Flow cytometer assay.

Flow cytometer analysis was performed as previously described (23, 52, 53). Briefly, bone marrow cells were harvested from femur and tibia with PBS. The spleen was minced and homogenized using the frosted ends of the slides and suspended in PBS. All the cells were passed through a 40 μm cell strainer to obtain single-cell suspension. ACK lysis buffer (Thermo Fisher Scientific A1049201) was applied to remove red blood cells when necessary. The preparation of peripheral blood was performed following previous studies (52, 54). The cells were then stained with appropriate antibodies at room temperature for 15–30 minutes, washed with PBS, and kept on ice until further analyses. Propidium iodide was added before the assay to exclude the dead cells. The gating strategies for hematopoietic stem and progenitor cells and MDSCs (CD11b^+^Ly6G^+^Ly6C^lo^, granulocytic MDSCs; and CD11b^+^Ly6G^−^Ly6C^hi^, monocytic MDSCs) have been described elsewhere (23, 52).

### Treatment of mice to target IL-6 signaling.

Anti-HRP IgG isotype control (BE0088, Bio X Cell), anti–mouse IL-6 monoclonal antibody (BE0046, Bio X Cell), and recombinant mouse gp130 Fc chimera protein (468-MG, R&D Systems) were diluted in *InVivo*Pure pH 7.0 dilution buffer (IP0070, Bio X Cell) to 10 μg/100 μL and separately injected once a week i.p. into old recipient mice that had undergone transplantation of old DKO bone marrow cells (10 μg/mouse). The treatment was started at 1 month after transplantation. Peripheral blood was collected retro-orbitally each month, and complete blood count of all mice was evaluated by Hemavet 950 (Drew Scientific).

For the chronic treatment, total bone marrow of 5-month-old DKO mice was collected. After red blood cell lysis, the cells were resuspended in PBS, and 1 × 10^6^ cells were then transplanted into 5-month-old CD45.1 lethally irradiated (10 Gy) recipient mice through retro-orbital injection. Anti-HRP IgG isotype control, anti–mouse IL-6 antibody, and gp130 Fc chimera protein were diluted as described above and separately injected i.p. into the above-mentioned DKO transplantation mice once a week (10 g/mouse). The treatment was started at 1 month after transplantation.

### ELISA and multiplex ELISA.

MDS patient bone marrow aspirate samples were resuspended in 5 mL of RPMI 1640 containing 100 U/mL preservative-free sodium heparin, 100 U/mL penicillin, and 100 μg/mL streptomycin. The supernatants were collected after centrifuging. ELISA was performed based on the manufacturer’s protocol to determine the expression levels of soluble IL-6R. Briefly, the bone marrow supernatants were diluted 50 times and incubated in antibody-precoated 96-well plates, together with human sIL-6R standard and empty controls. After mixing with HRP conjugate, the plate was incubated at room temperature for 2 hours. After incubation, the plates were washed with washing buffer 3 times, followed by addition of TMB substrate solution in each well and incubation for 20 minutes at room temperature. The reaction was terminated by pipetting of stop solution to each well, and the absorption at 450 nm wavelength was measured in a spectrophotometer. All samples were performed in duplicate in the assay. The same experiment was done for the detection of mouse sIL-6R. Mouse and human sIL-6R ELISA kits were purchased from MyBioSource (MBS722764) and Thermo Fisher Scientific (BMS214), respectively.

The Mouse Magnetic Luminex Screening Kit, detecting over 40 cytokines, was purchased from R&D Systems (LXSAMSM-44). The Luminex assay was performed at the Comprehensive Metabolic Core of Northwestern University. The mean fluorescence intensity of each sample was calculated and analyzed. The similarity matrix and hierarchical clustering with Pearson correlation were obtained through the online tool Morpheus (https://software.broadinstitute.org/morpheus/).

### Single-cell RNA sequencing.

Single bone marrow mononuclear cells from aged mice were applied to the 10x Genomics platform for single-cell RNA sequencing at the NUSeq core facility of Northwestern University. An estimated 10,000 cells were loaded into the 10x Chromium system per sample. RNA was converted to cDNA, and libraries were generated using the Chromium Single Cell 3′ Kit v3. A Bioanalyzer (Agilent) confirmed that the size of the main peaks of cDNA was generally between 450 and 490 bp. The prepared libraries were sent to BGI (Hong Kong) with DNBseq PE 100 platform for sequencing. The sequencing data were processed (including alignment and quantification) using the Cell Ranger pipeline. The joint analysis of 3 single-cell data sets was performed by Seurat v4. The percentage of mitochondrial and ribosomal genes per cell was calculated and added to the metadata. The proportion of hemoglobin genes was examined to eliminate red blood cell contamination. Filtering criteria “min. cells = 3, min. features = 200” were applied to filter the preliminary data. Single cells with fewer than 500 detected genes or more than 4000 detected genes or more than 15% reads aligned to mitochondrial genes were further excluded. For cell clustering analysis, the IntegrateData function in the Seurat package was used for data combination with resolution value = 0.5 and 30 principal components. The Uniform Manifold Approximation and Projection (UMAP) algorithm was adopted to perform nonlinear dimensionality reduction analysis and cell clustering. The cluster-specific marker genes were screened by calculation of differentially expressed genes (DEGs) in each cluster compared with all remaining cells, and the cell type for each cluster was annotated and re-marked with the CellMarker database (http://biocc.hrbmu.edu.cn/CellMarker/index.jsp). DEG analysis was further performed between DKO and TWT or between DKO and TKO samples among the identical cluster. Pathway enrichments were identified according to the KEGG annotation and clarification. Single-cell RNA sequencing data were uploaded to the NCBI’s Gene Expression Omnibus database (GEO GSE206600).

### Histology staining.

Mouse sternum, spleen, liver, and lung were fixed in 10% neutral-buffered formalin overnight. The samples were then embedded in paraffin and processed for H&E staining at the Mouse Histology and Phenotyping Laboratory of Northwestern University. Peripheral blood or bone marrow smears were stained with May-Grünwald-Giemsa staining as previously described (6, 23).

### Xenograft of MDSL cells in NSG mice.

NOD/SCID-IL2Rγ (NSG) mice were purchased from The Jackson Laboratory (stock 005557). MDSL cells (1 × 10^6^) were retro-orbitally injected into 12-week-old sublethally irradiated (2.5 Gy) female NSG mice. Ten days after transplantation, human IgG isotype control (BE0297, InVivoMAb) or tocilizumab (Selleck) was administrated at 8 mg/kg weekly via i.p. injection. To evaluate the engraftment, peripheral blood was collected from tail vein on day 60. The peripheral blood mononuclear cells were stained with anti–human CD45 (368521, BioLegend) after red blood cell lysis (RBC Lysis Buffer, eBioscience) for flow cytometric analysis.

### Colony-forming unit assay.

Patient bone marrow–derived CD34^+^ cells were isolated using a human CD34 MicroBeads Kit (130-046-702, Miltenyi Biotec) and MACS Magnetic Separators following the manufacturer’s instruction. In brief, mononuclear cells from 10 mL of total bone marrow aspirate were separated using density gradient centrifugation with Ficoll-Paque buffer (ρ = 1.077 g/mL). Mononuclear cells were incubated with CD34 MicroBeads and FcR Blocking Reagent at 4°C for 30 minutes before magnetic column separation. Collected CD34^+^ cells were then aliquoted to 0.3 × 10^6^ per vial and stored in liquid nitrogen. Frozen normal human bone marrow CD34^+^ cells were purchased from StemCell Technologies.

For colony-forming unit (CFU) assay, CD34^+^ cells were thawed in a 37°C water bath. Cells were spun down and resuspended in IMDM without FBS. Cell viability was assessed with trypan blue staining and counted with a Bio-Rad TC20 cell counter. Live normal CD34^+^ cells (4 × 10^3^) were mixed with 4 mL of MethoCult Optimum medium (H4034, StemCell Technologies) supplemented with IgG or tocilizumab at a final concentration of 50 μg/mL. Given the potentially lower number of colonies generated from MDS patient CD34^+^ cells (55), 8 × 10^3^ live patient CD34^+^ cells were mixed with 4 mL of MethoCult Optimum medium supplemented with human IgG isotype control or tocilizumab at a final concentration of 50 μg/mL. The cell suspension was vigorously mixed by vortexing, and subsequently aliquoted at 1 mL per well in 6-well plates. The vacant wells were filled with distilled water to prevent the MethoCult from drying out. Fourteen days after seeding, the CFU assay was ready for evaluation. Colonies were counted under an inverted microscope (EVOS M5000, Thermo Fisher Scientific), and the colonies were identified by 2 independent individuals.

### Testing of IL-6 signaling.

Mononuclear cells were obtained from wild-type bone marrow cells after RBC lysis (eBioscience). Cells were resuspended in serum-free RPMI 1640 medium; 1 × 10^6^ cells were then seeded in each well of a 12-well plate. Cells were treated with anti–mouse IL-6 antibody (BE0046, InVivoMAb) or mouse gp130 Fc chimera for 1 hour under culture condition. Mouse IgG1 isotype control and control Fc fusion protein (Enzo Life Sciences) were used as negative controls for anti–mouse IL-6 antibody and mouse gp130 Fc chimera, respectively. Cells were challenged with mouse recombinant IL-6 at a final concentration of 10 ng/mL for 15 minutes and then harvested with RIPA buffer for Western blot analyses.

### Statistics.

Results are expressed as mean ± SEM or mean ± SD, as indicated. The statistical analysis was performed with 2-tailed Student’s *t* test, 1-way ANOVA with Tukey’s multiple-comparison test, or 2-way ANOVA with Tukey’s multiple-comparison test using GraphPad Prism v8.0 software. Survival curves were compiled using Kaplan-Meier algorithms of Prism software, and significance was assessed using the log-rank (Mantel-Cox) test. *P* less than 0.05 was considered statistically significant.

### Study approval.

MDS patient samples were obtained from leftover diagnostic specimens at the Department of Pathology, Northwestern University. The study protocol was approved by the institutional review board at Northwestern University.

All the animal experiments were conducted in accordance with the *Guide for the Care and Use of Laboratory Animals* (National Academies Press, 2011) and approved by the Institutional Animal Care and Use Committee at Northwestern University.

## Author contributions

YM, KR, YL, XH, EL, HB, and JY performed the experiments and interpreted data. HT, AV, XL, YA, JKA, MS, and PJ obtained and interpreted the clinical data. YM, AM, ZX, XX, CZ, DZ, ZL, and LD analyzed the single-cell RNA sequencing data. YM, KR, and PJ designed the experiments, interpreted data, and wrote the manuscript. The order of the co–first authors was determined based on their efforts and contributions to the manuscript.

## Supplementary Material

Supplemental data

## Version 1 07/28/2022

In-Press Preview

## Version 2 07/28/2022

In-Press Preview

## Version 3 09/01/2022

Electronic publication
