# Supplementary material for: Bone marrow–confined IL-6 signaling mediates the progression of myelodysplastic syndromes to acute myeloid leukemia
Source: J Clin Invest. 2022 Sep 1;132(17):e152673. doi: 10.1172/JCI152673 (PMC9435651; doi:10.1172/JCI152673)

Supplemental Data

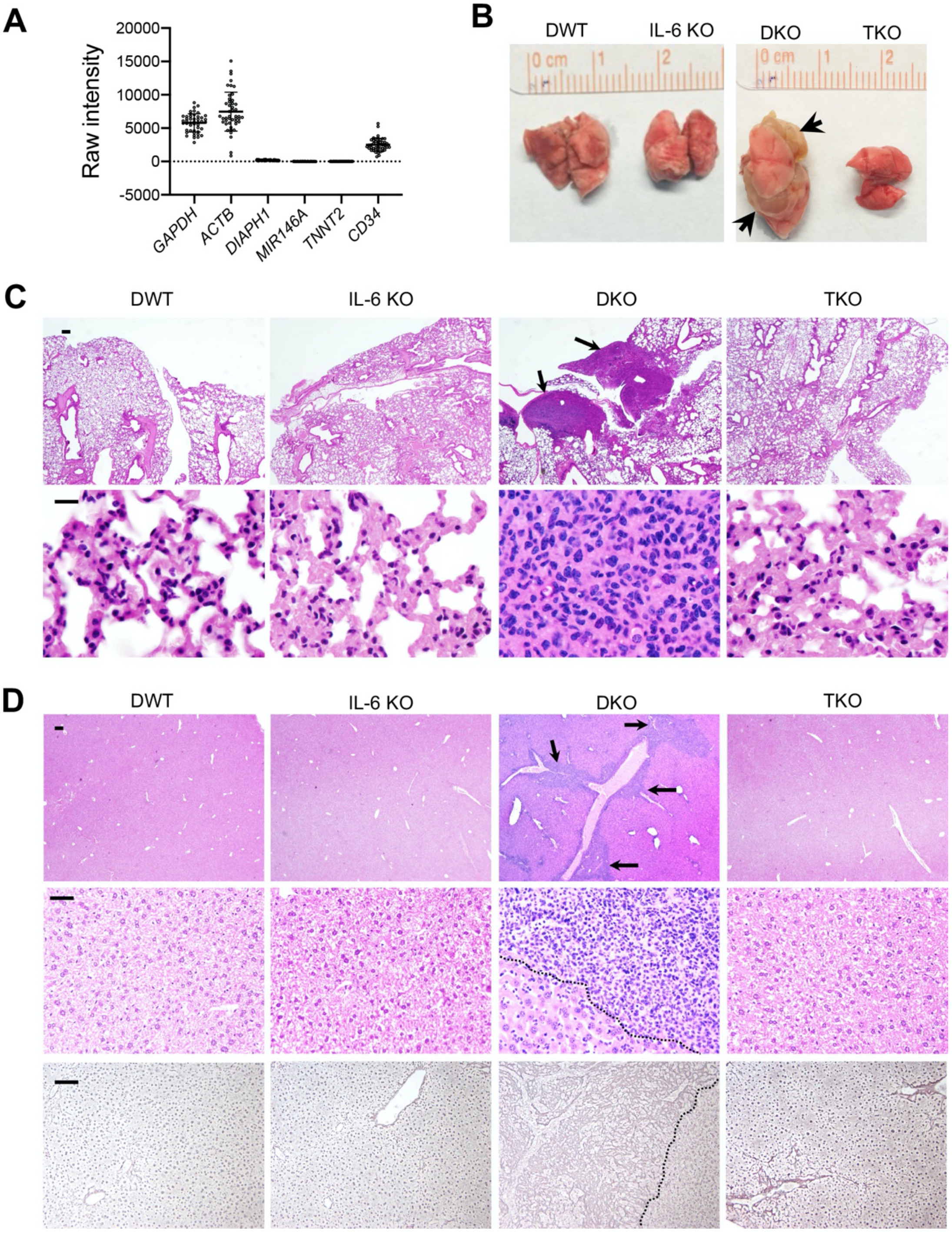

Supplemental Figure 1. Loss of IL-6 rescues multi-organ leukemia infiltrations in DKO mice. (A) Microarray gene expression data of bone marrow CD34+ cells from 47 MDS patients. Housekeeping genes GAPDH and ACTB were plotted to demonstrate the modest raw intensity

without normalization. TNNT2 and CD34 were plotted as negative and positive controls, respectively. Data is from GSE160727. (**B**) Representative gross images of the lungs from the indicated mice at the age of 12-14 months. Arrows indicate tumors. **(C)** Representative images of H&E staining of the lung tissues from A. Arrows indicate the blasts involvement in DKO mice, which was illustrated in the image below. Scale bars: 100 µm. **(D)** Representative images of H&E staining and reticulin staining (bottom row) of the liver tissues from indicated mice at the age of 12-14 months. Arrows in the DKO panel indicate blast infiltration. The dotted lines on DKO panels separate normal and neoplastic tissues. Scale bar: 100 µm.

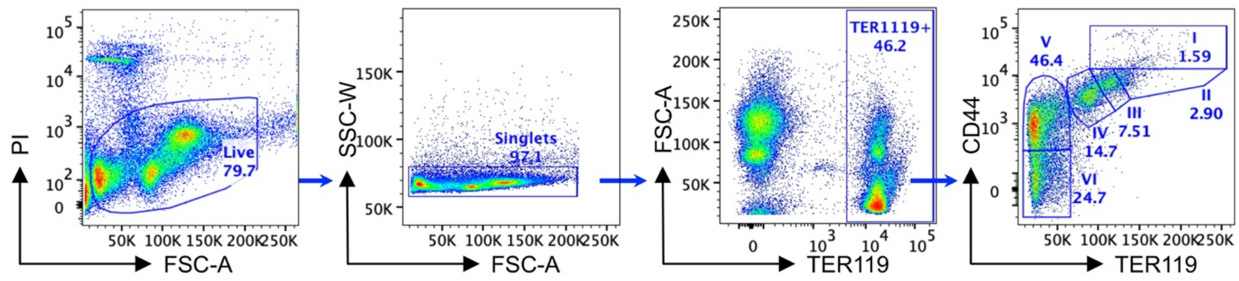

Supplemental Figure 2. Gating strategy for the identification of different erythroblast populations.

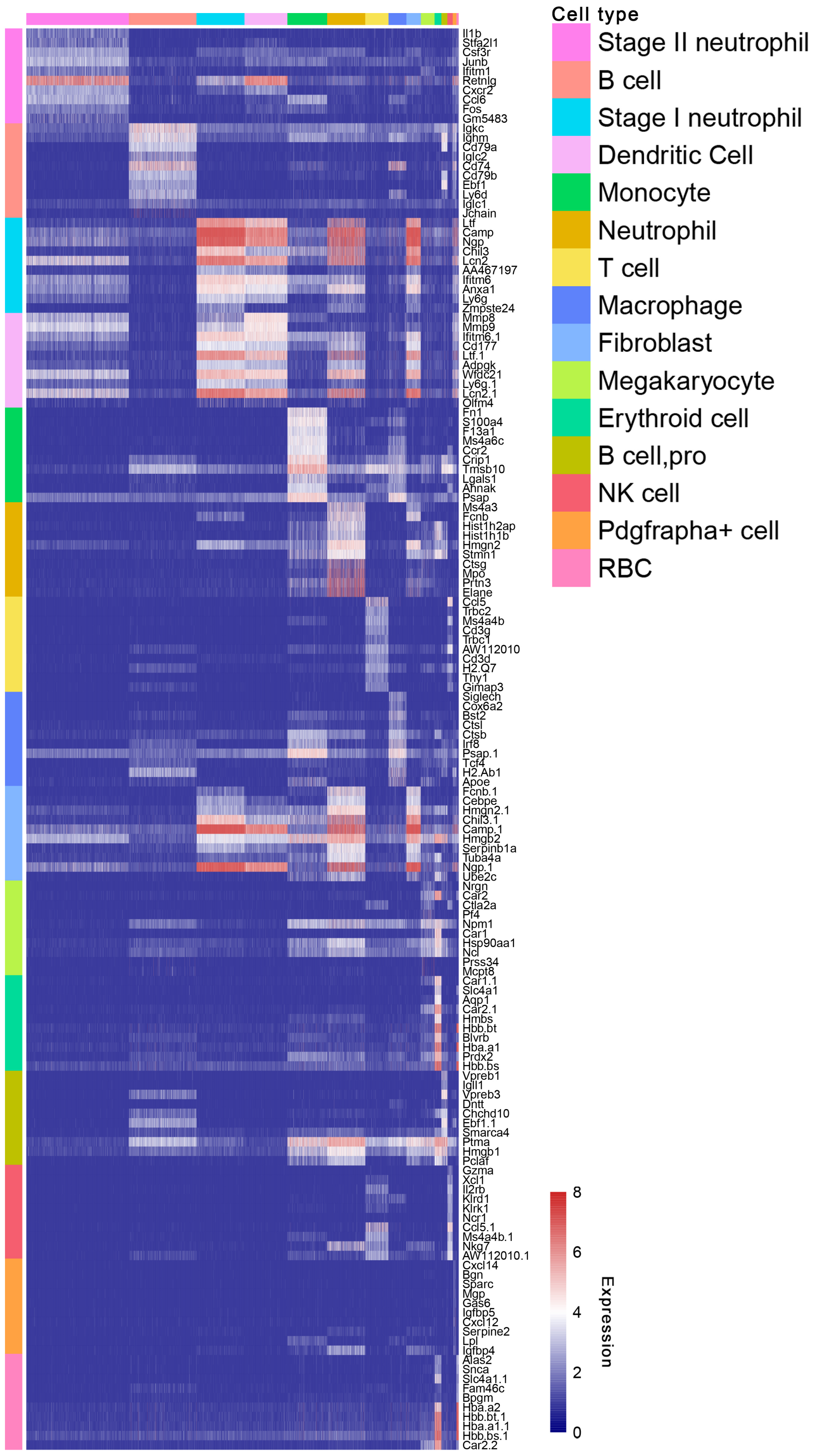

Supplemental Figure 3. Maker genes for each identified cell population in TWT mice in Fig. 4A-D were clustered and shown in heatmap.

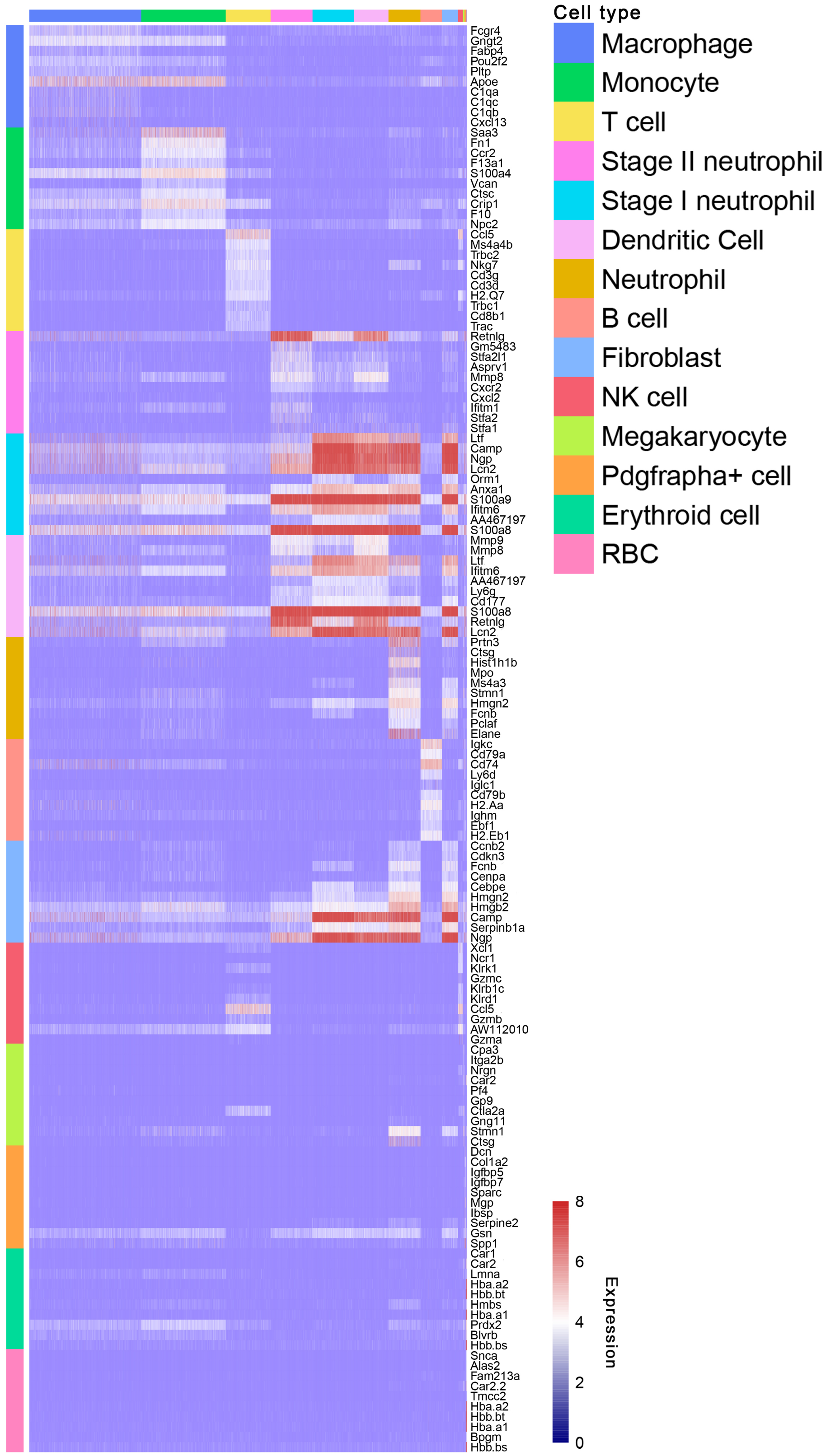

Supplemental Figure 4. Maker genes for each identified cell population in DKO mice in Fig. 4A-D were clustered and shown in heatmap.

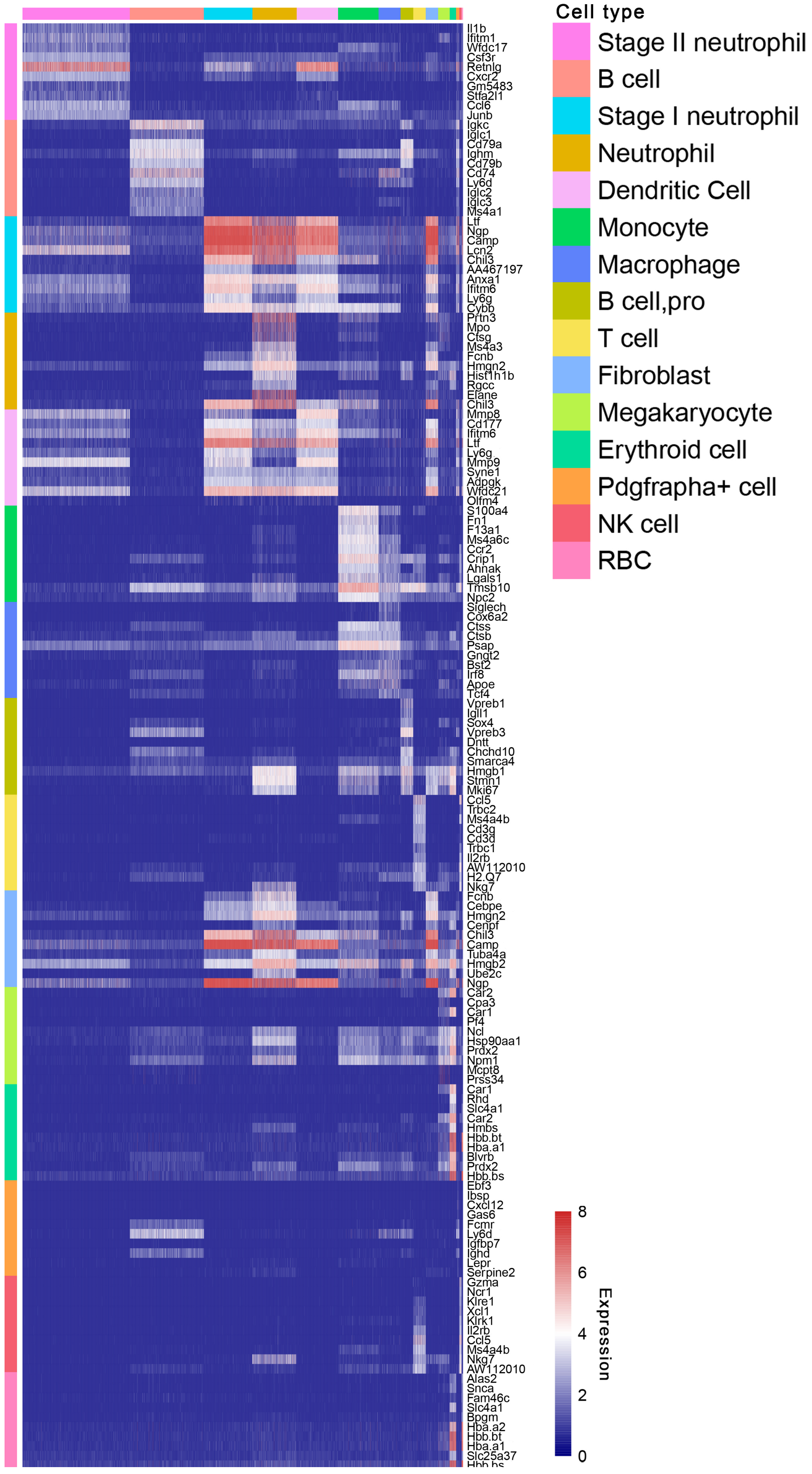

Supplemental Figure 5. Maker genes for each identified cell population in TKO mice in Fig. 4A-D were clustered and shown in heatmap.

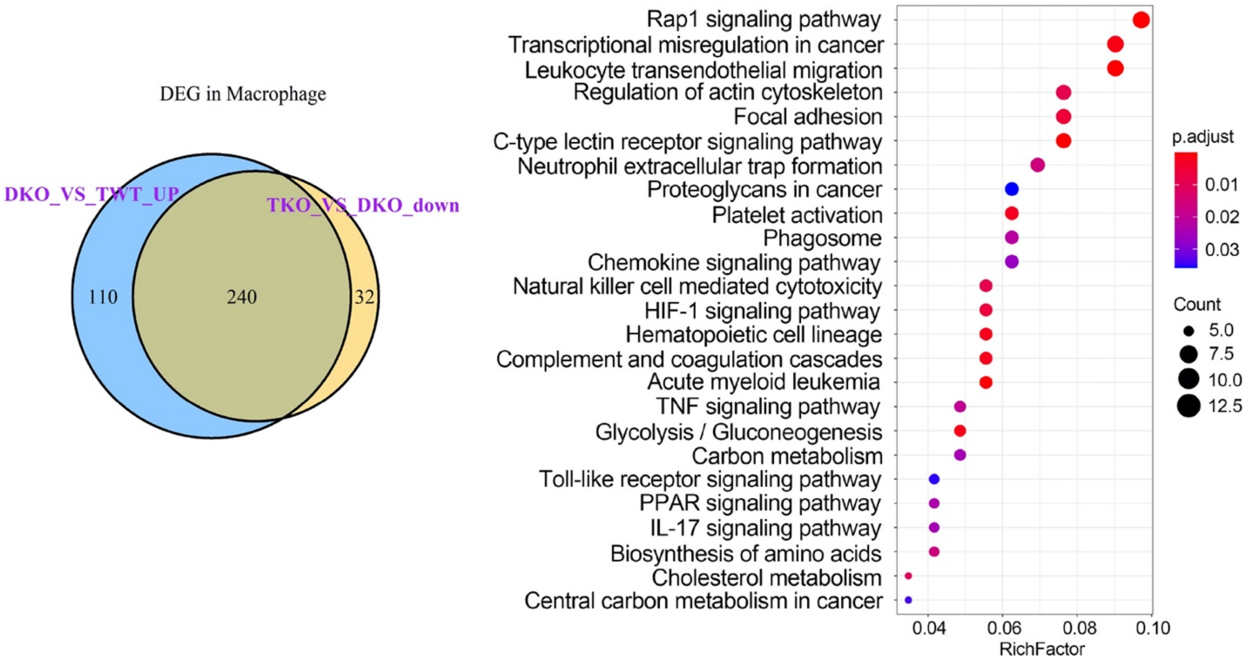

Supplemental Figure 6. Loss of IL-6 reverted genes that are upregulated in the macrophage group in DKO bone marrow. Differentially expressed genes (DEG) in macrophage group were illustrated in Venn diagrams. KEGG pathway analyses of common DEG are on the right.

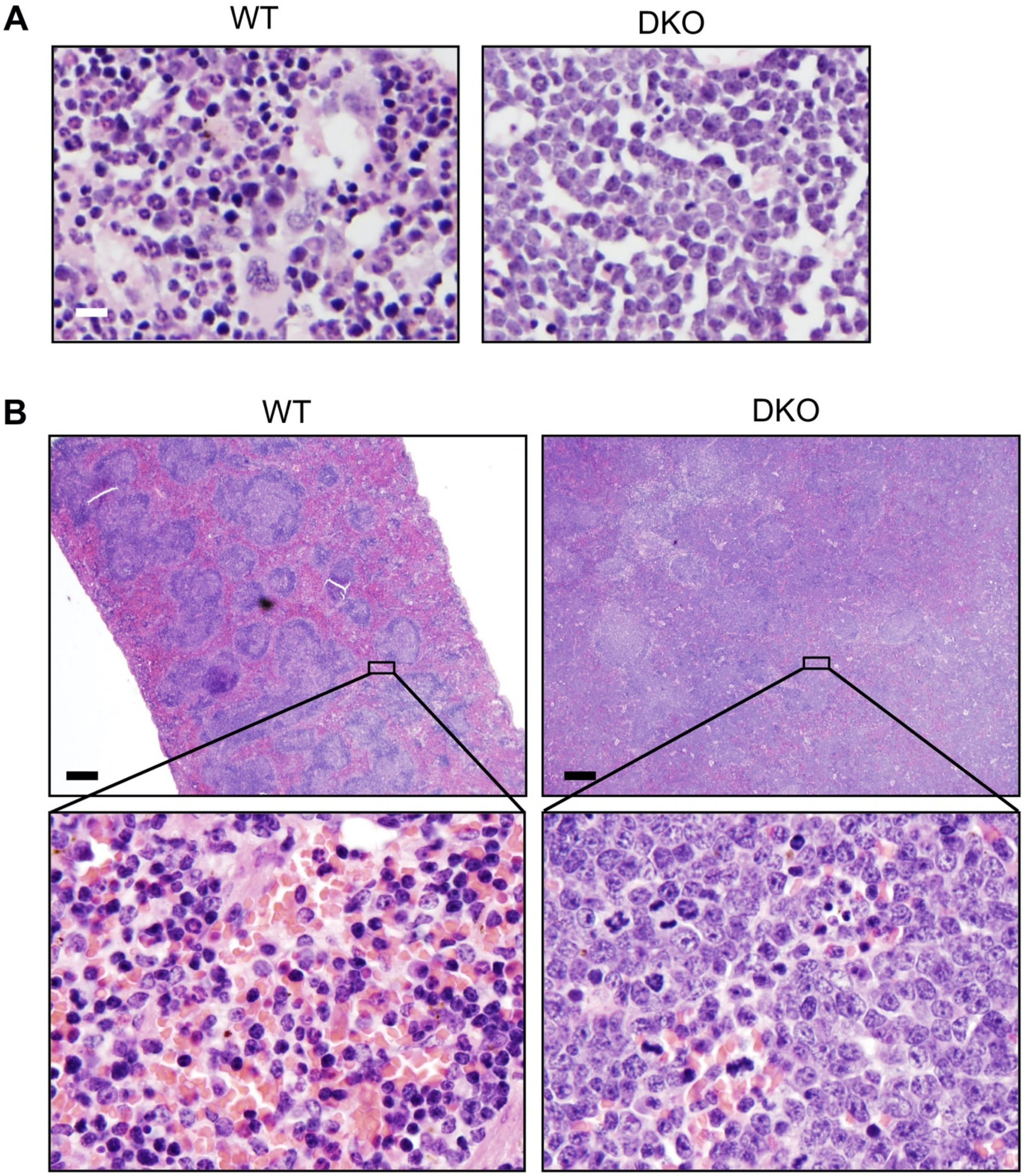

Supplemental Figure 7. The transplantable leukemia from DKO mice is derived from the c-Kit positive cells. (**A**) Representative bone marrow sections of recipient mice at 5 months after transplantation of spleen c-Kit+ cells from 12-month-old DKO mice. Mice in the WT control group were transplanted with bone marrow c-Kit+ cells from 12-month-old TWT mice. Scale bar: 20 µm. (**B**) Same as A except spleen sections were analyzed in the indicated mice. H&E staining, scale bars: 100 µm.

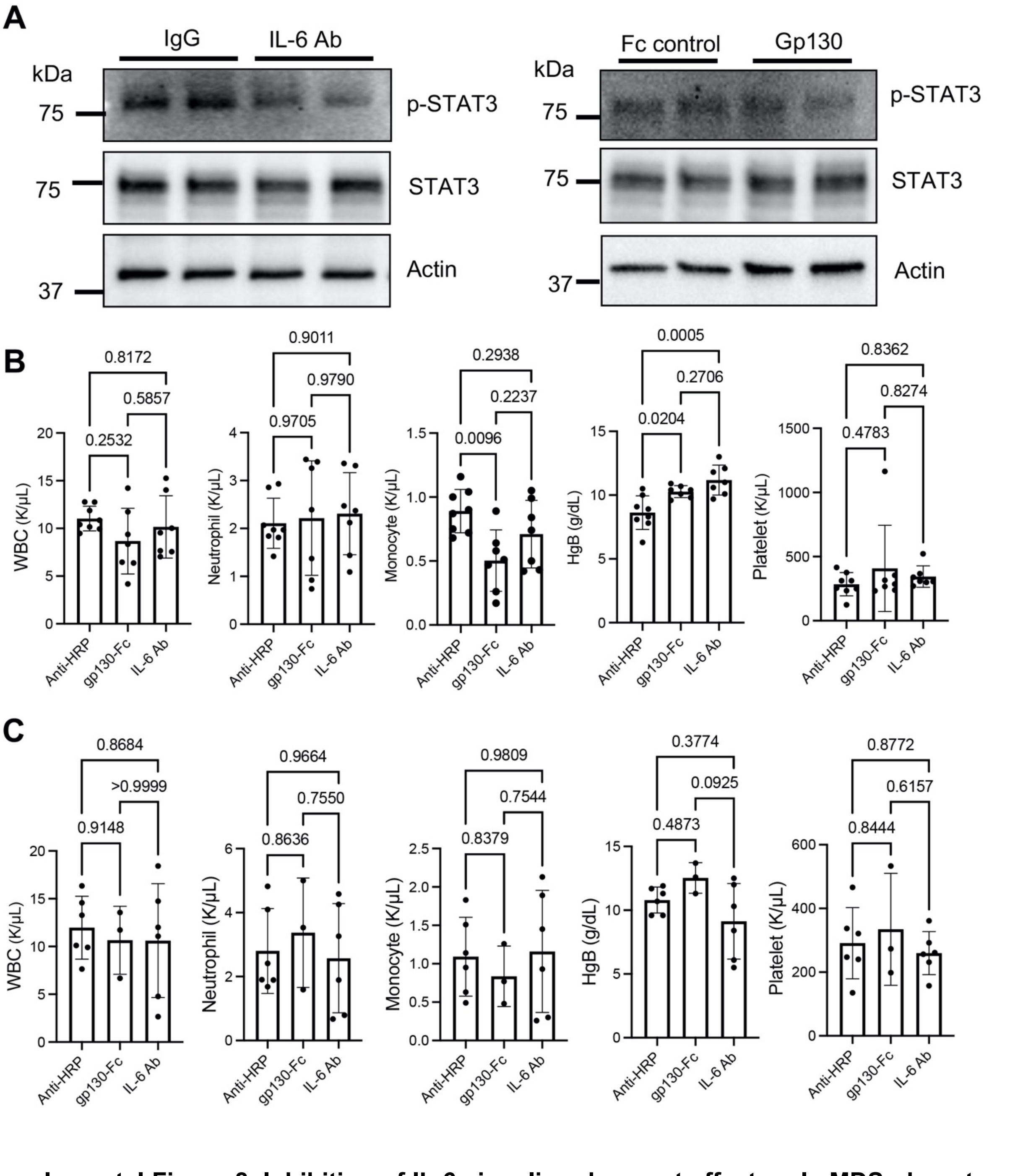

Supplemental Figure 8. Inhibition of IL-6 signaling does not affect early MDS phenotypes in DKO mice. (A) Total bone marrow cells from wild-type C57/BL6 mice were collected in serum-free RPMI1640 medium with anti-mouse IL-6 antibody (left) or mouse gp130-Fc chimera (right). After 1 hour incubation, cells were challenged with mouse recombinant IL-6 for 15 minutes before RIPA lysis for Western blot analyses of the indicated proteins. **(B-C)** 5-month-old recipient mice were transplanted with bone marrow cells from 5-month-old DKO mice. The recipient mice were treated with Anti-HRP IgG control, gp130-Fc chimera, or anti-IL-6 antibody once every week. Complete blood counts were analyzed at 1 month (B) and 4 month (C) post-treatment. Data presented in Mean ± SD. P values are listed above the plots.

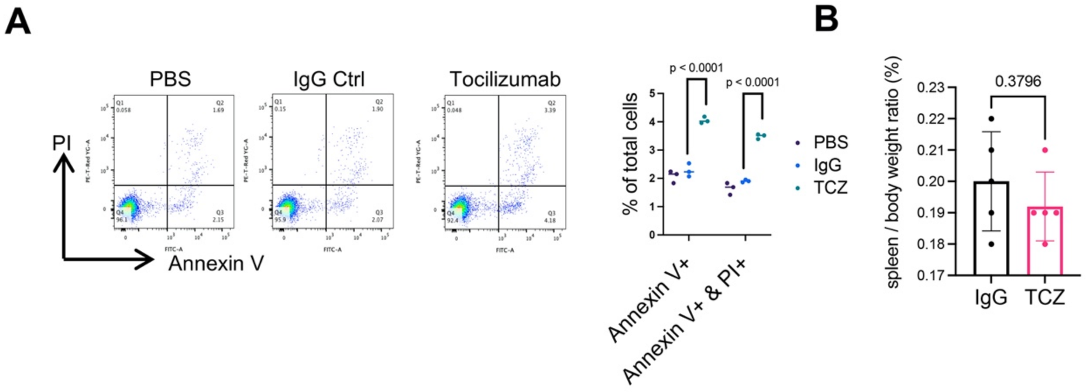

Supplemental Figure 9. Tocilizumab partially induces cell death in MDSL cells (A) and reduces spleen weight in MDSL xenograft model (B).

Supplemental Tables.

Supplemental Table 1. The clinical information of patients with high risk MDS

	Patient #1	Patient #2	Patient #3	Patient #4
**Age (year)**	80	74	66	69
**Gender**	Female	Male	Male	Female
**Diagnosis**	Myelodysplastic syndrome with multilineage dysplasia (MDS-MLD)	High grade myeloid neoplasm	Myelodysplastic syndrome with excess blasts-2 (MDS-EB-2)	Myelodysplastic syndrome with excess blasts-2 (MDS-EB-2)
**Bone marrow blasts**	2%	15-20%	10%	15%
**Next generation sequencing**	*ASXL1* , *PHF6* , and *TET2* mutations	*ASXL1* , *SRSF2* , *SETBP1* , and *KRAS* mutations	Two *TP53* mutations	*IDH2* and *BCOR* mutations
**Karyotype**	46,XX[20]	47,XY,+13,+17,del(17)(p13p11.2),del(12)(p13p12),dic(17;20)(p11.2;q11.2)[cp6]	48~50,XY,add(3)(q12),der(5)t(5;15)(q15;q11.2),+6,add(6)(q23),del(7)(q22q36),+8,del(9)(p24p13),der(11)add(11)(p11.1)del(11)(q13q21),-13,-15,-19,+3~6mar[cp18]	47,XX,+4[2]

Supplemental Table 2. Antibodies used for flow cytometric assays and immunohistochemistry staining.

Antibodies for flow cytometric assay		
Antibody	Catalog number	Supplier or comments
PE-CD11b	M1/70, Cat# 553311	BD Pharmingen
PE-Cy7-Gr1	RB6-8C5, Cat# 108416	BioLegend
Pacific Blue-B220	RA3-6B2, Cat# 103227	BioLegend
APC-eFluor780-CD3e	17A2, Cat# 47-0032-82	eBioscience
APC/Fire 750-CD3e	17A2, Cat# 100248	BioLegend
PE-Cy7-Ly6G	1A8, Cat# 560601	BD Pharmingen
APC-Ly6C	HK1.4, Cat# 128016	Biolegend
APC-TER-119	TER-119, Cat# 17-5921-82	eBioscience
PE-CD44	IM7, Cat# 12-0441-82	eBioscience
BV421-CD34	RAM34, Cat# 562608	BD Horizon
PE-Sca1(Ly-6A/E)	D7, Cat# 108108	BioLegend
PE-Cy7-CD117(c-Kit)	2B8, Cat# 105814	BioLegend
Pacific Blue-CD117(c-Kit)	2B8, Cat# 105820	BioLegend
APC-CD117(c-Kit)	2B8, Cat# 17-1171-82	eBioscience
APC anti-mouse CD126 (IL-6Rα chain)	D7715A7, Cat# 115812	Biolegend
BV421 anti-human CD45	2D1, Cat# 368521	Biolegend
Antibodies for immunohistochemistry staining		
Anti-Human IL-6R	Cat # PA5-102425	Invitrogen
Antibodies for Western blotting		
Anti-Stat3 rabbit mAb	Cat# 12640	Cell Signaling Technology
Anti-pStat3 (Tyr705) rabbit mAb	Cat# 9145	Cell Signaling Technology
Anti-beta actin mouse mAb	Cat# 12262	Cell Signaling Technology

Figure 8A:

p-STAT3

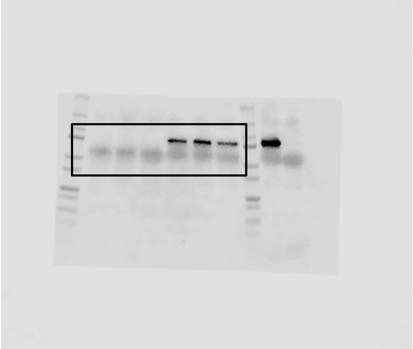

Actin

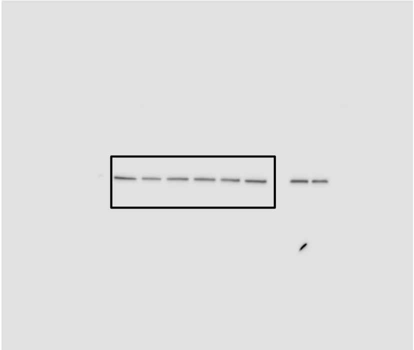

Supplemental Figure 6A left panel:

p-STAT3

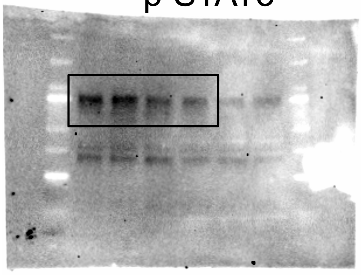

STAT3

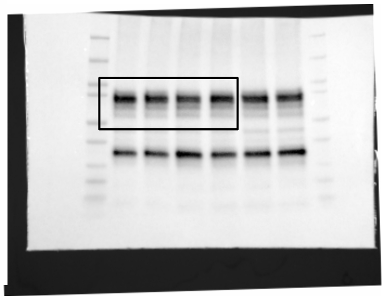

Actin

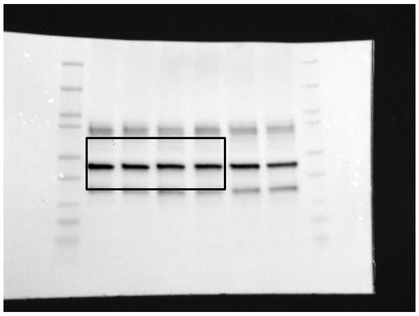

Supplemental Figure 6A right panel:

p-STAT3

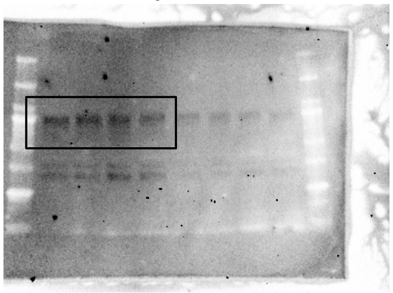

STAT3

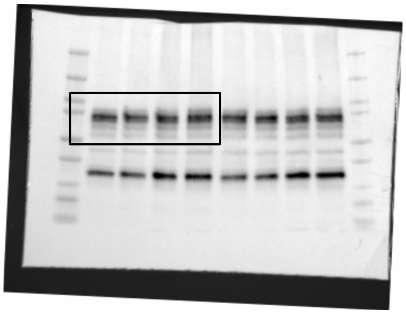

Actin

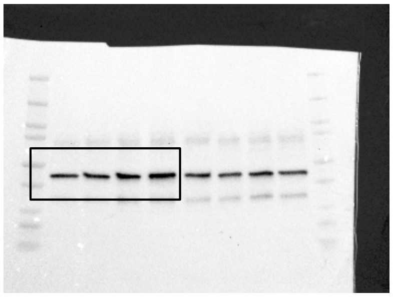

Supplement: Supplemental data [file jci-132-152673-s098.pdf]
